# The Relationship of COVID-19 Vaccination with Mortality Among 86,732 Hospitalized Patients: Subpopulations, Patient Factors, and Changes over Time

**DOI:** 10.1007/s11606-022-08007-0

**Published:** 2023-01-18

**Authors:** Timothy B. Baker, Daniel M. Bolt, Stevens S. Smith, Thomas M. Piasecki, Karen L. Conner, Steven L. Bernstein, Todd Hayes-Birchler, Wendy E. Theobald, Michael C. Fiore

**Affiliations:** 1 Center for Tobacco Research and Intervention, School of Medicine and Public Health, University of Wisconsin School of Medicine and Public Health, 1930 Monroe St #200, Madison, WI 53711 USA; 2 Department of Medicine, School of Medicine and Public Health, University of Wisconsin-Madison, Madison, WI USA; 3 Department of Educational Psychology, University of Wisconsin-Madison, Madison, WI USA; 4 Department of Emergency Medicine, Geisel School of Medicine at Dartmouth, Lebanon, NH USA

**Keywords:** COVID, vaccination, COVID mortality risk factors, change in COVID mortality over time

## Abstract

**Background:**

Information on COVID-19 vaccination effects on mortality among patients hospitalized with COVID-19 could inform vaccination outreach efforts and increase understanding of patient risk.

**Objective:**

Determine the associations of vaccination status with mortality in adult patients hospitalized with COVID-19.

**Design:**

This retrospective cohort study assessed the characteristics and mortality rates of adult patients hospitalized with COVID-19 across 21 healthcare systems in the USA from January 1, 2021, to January 31, 2022.

**Participants:**

Adult patients admitted to participating hospitals who had COVID-19 diagnoses and/or positive PCR tests and completed their hospital stay via discharge or death.

**Main Measure:**

In-hospital mortality vs. discharge (outcome) and patient age, sex, race, ethnicity, BMI, insurance status, comorbidities, and vaccination status extracted from the electronic health record (EHR).

**Key Results:**

Of 86,732 adult patients hospitalized with COVID-19, 45,082 (52%) were female, mean age was 60 years, 20,800 (24%) were Black, and 22,792 (26.3%) had one or more COVID-19 vaccinations. Statistically adjusted mortality rates for unvaccinated and vaccinated patients were 8.3% (95% CI, 8.1–8.5) and 5.1% (95% CI, 4.8–5.4) respectively (7.9% vs. 4.5% with no immune compromise). Vaccination was associated with especially large reductions in mortality for obese (OR = 0.67; 95% CI 0.56–0.80) and severely obese (OR = 0.52; 95% CI, 0.41–0.67) patients and for older patients (OR = 0.99; 95% CI, 0.98–0.99). Mortality likelihood was higher later in the study period (August 2021–January 31, 2022) than earlier (January 1, 2021–July 30, 2021) (OR = 1.10; 95% CI = 1.04–1.17) and increased significantly for vaccinated patients from 4.6% (95% CI, 3.9–5.2%) to 6.5% (95% CI, 6.2–6.9%).

**Conclusions:**

Patients vaccinated for COVID-19 had reduced mortality, especially for obese/severely obese and older individuals. Vaccination’s protective effect against mortality declined over time and hospitalized obese and older individuals may derive especially great benefit from prior vaccination against SARS-CoV-2.

**Supplementary Information:**

The online version contains supplementary material available at 10.1007/s11606-022-08007-0.

COVID-19 has disrupted virtually every aspect of society, infecting over 85 million individuals in the USA and causing over one million COVID deaths through June 2022.^1^ Vaccination with any of the FDA-approved SARS-CoV-2 (COVID-19) vaccinations can prevent more severe COVID-19 disease,^2–4^ protect against different COVID-19 variants,^2–5^ and produce persistent effects.^2^ However, important knowledge gaps remain.

Little research has examined vaccination effects in hospitalized populations, patients that typically have the most severe COVID disease. While some studies track individuals as they transition from nonhospitalized to hospitalization status,^2,3,6–9^ there is less research on vaccination effects in large, hospitalized samples. Research with relatively small samples of hospitalized patients shows that vaccination reduces mortality.^4,10,11^ However, the small sample sizes of these studies limit the ability to determine associations between vaccination and disease severity in specific patient groups. Such information could reveal groups who would benefit from additional preventive or ameliorative actions to reduce their risk of COVID-19 morbidity or mortality.

This study examined associations between COVID-19 vaccination status and mortality in a sample of 86,732 patients who were hospitalized with COVID-19 from January 2021, when COVID-19 vaccination became generally available, to January 2022.

## METHODS

### Study Design

**Figure 1:**
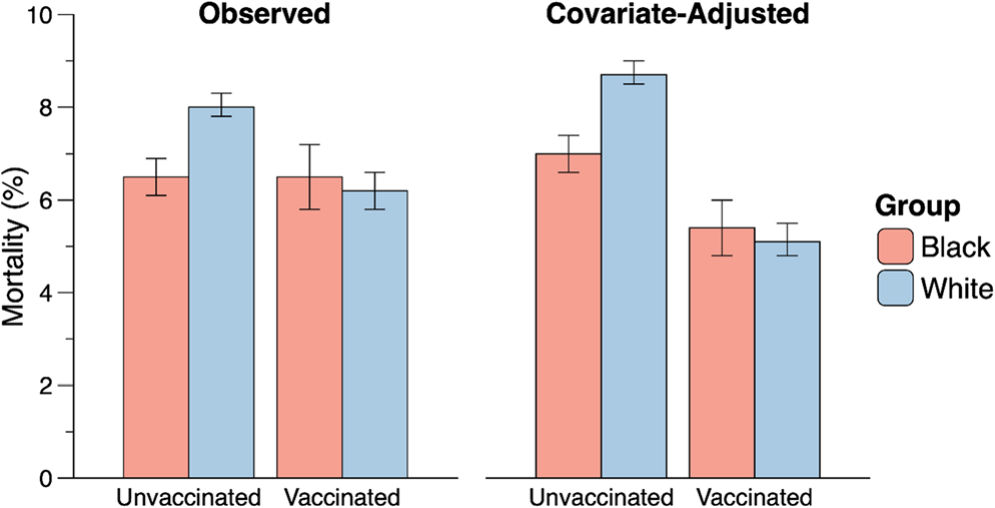
Mortality rates among Black and White patients hospitalized with COVID-19 as a function of vaccination Status (binary).

The COVID EHR Cohort at the University of Wisconsin (CEC-UW) is a retrospective cohort study funded by the National Cancer Institute (NCI). Health systems from across the USA were invited to participate; 21 joined the cohort (S Figure 1) and transferred data regularly to the CEC-UW Coordinating Center in Madison, Wisconsin. Each data extraction was retrospective to January 2021 for the analysis sample and captured both new patients entering the cohort and follow-up data from patients identified at earlier extractions.

### Ethics Statement

The CEC-UW study was initially approved in May 2020 by the University of Wisconsin-Madison Health Sciences Minimal Risk Institutional Review Board (MR-IRB) for collection of de-identified EHR data. In February 2021, the MR-IRB approved a protocol change to a Limited Data Set.

### Data Collection

#### Extraction, Harmonization, and Secure Transfer of EHR Data

EHR data extraction code was created by programmers at UW School of Medicine and Public Health (Madison, WI), Yale New Haven Health (New Haven, CT), and Bluetree Network, Inc. (now Tegria).^12^

Data elements were extracted from defined fields for patient sociodemographic variables, general health information, clinical encounter data, pre-COVID and post-COVID ICD-10 diagnoses, laboratory test results, and medication information (S Methods).

The extraction code was customized at each health system to map to their EHR data to yield relatively uniform data sets. Additional data harmonization and quality assurance were done by CEC-UW data operations staff (S Methods). Secure transfer of data from each of the 21 health systems was accomplished via the transfer of data files to a secure SFTP (secure shell [SSH] File Transfer Protocol) portal located at the UW-Madison CEC-UW Coordinating Center.

### Extracted Data Categories

The five categories of data extracted (i.e., demographics, ICD-10 diagnoses, clinical encounter data, laboratory tests, and medications) are described in the S Methods. Health systems provided data only for closed clinical encounters (i.e., completed). For closed inpatient encounters, the patient must have been discharged (or transferred) or died during the hospitalization. Data on outcomes or treatment at nonparticipating health systems were not captured.

### Analysis Sample

The analysis sample comprised 86,732 adult patients hospitalized with COVID-19 who were admitted to a participating hospital and completed their hospitalization over the period from January 1, 2021, to January 31, 2022 (Table 1). Analysis sample inclusion criteria included the following: (1) age ≥18 years old; (2) the inpatient encounter was the first COVID hospitalization with duration ≥ 24 h (or, if < 24 h, admission to ICU or death during the hospitalization); and (3) prior contact with the health system to permit extraction of vaccination data and pre-COVID ICD-10 diagnoses to calculate the Elixhauser Comorbidity Index score^13^ (S Methods). In addition, positive COVID-19 status was determined by either COVID ICD-10 diagnosis (U07.1 or J12.82) during the hospitalization or a positive COVID PCR test result in a 14-day window (± 7 days centered at the admission date).

**Table 1:** Descriptive Statistics for 86,732 Hospitalized COVID-19 Patients from January 2021 to January 2022

Patient characteristic	*N*	%	*M*	SD
Age groups				
Under 60 years	38,775	44.7		
60–70 years	19,593	22.6		
Over 70 years	28,364	32.7		
Sex				
Female	45,082	52.0		
Male	41,649	48.0		
Other	1	<0.01		
Race				
American Indian/Alaska Native	323	0.4		
Asian	2065	2.4		
Black or African American	20,800	24.0		
Native Hawaiian or other Pacific Islander	269	0.3		
White	53,017	61.1		
Other race	8470	9.8		
More than one race	359	0.4		
Missing	1429	1.6		
Ethnicity				
Not Hispanic or Latino	74,222	85.6		
Hispanic or Latino	10,901	12.6		
Missing	1609	1.9		
Body mass index				
Underweight	2767	3.2		
Healthy weight	20,100	23.2		
Overweight	24,128	27.8		
Obese	28,620	33.0		
Severely obese	10,299	11.9		
Missing	818	0.9		
Insurance status				
Medicare	43,482	50.1		
Medicaid	10,790	12.4		
Commercial	24,032	27.7		
Uninsured	2046	2.4		
Other	6382	7.4		
Vaccination status				
No recorded vaccination	63,940	73.7		
Yes, at least one	22,792	26.3		
Vaccination doses				
0	63,940	73.7		
1	5569	6.4		
2	13,647	15.7		
3	3576	4.1		
Elixhauser Comorbidity Index			6.0	10.1
Age (years)			60.0	18.6

### Primary Outcome

The primary and sole outcome for these analyses was in-hospital mortality during the index COVID hospitalization documented via EHR.

### Non-outcome Variables

Patient-level variables include age (at time of entry into the cohort), sex, race, ethnicity, body mass index (BMI), insurance status, Elixhauser Comorbidity Index score and items, and vaccination status. Preadmission vaccination status was coded either as binary (no vaccination versus any vaccination) or by the number of vaccine doses (0, 1, 2, or 3 doses). S Table 1 presents the types of vaccines that patients received. Patients aged ≥ 90 years were coded as 90 at the time of data extraction for HIPAA compliance. For certain analyses, age was categorized as follows: 18–59 years, 60–70 years, and over 70 years (cut-points suggested by class probability trees) (see Table 1 for race, ethnicity, BMI categories, and insurance status categories). Race and ethnicity categories were based on definitions used by the National Institutes of Health.^14^ The Elixhauser Comorbidity Index score was calculated using van Walraven weights^13^ (S Methods) based on ICD-10 diagnoses (present vs. absent) determined via a 5-year look back pre-COVID.

### Statistical Analysis

#### Descriptive Statistics and Missingness

Descriptive statistics for the analysis sample characteristics and selected outcome analyses were computed using R (R Core Team: https://www.R-project.org/). There were no missing data for the primary outcome. Covariate missingness is reported in Table 1.

#### Vaccination Rates over the Study Period

Analyses were conducted to determine the change in vaccination status over the study period. The outcome was the binary vaccination variable reflecting whether or not a patient had received any vaccination (coded 0=no vaccination; 1=any vaccination). The analyses used logistic regression to examine a linear effect of time (0–12) corresponding to month. These analyses examined the main effect of time and then the interactions between time and each of the 7 covariates. A final model included the main effect of time and its interaction with all covariates. Site effects were examined but not included since they were negligible.

### Predicting Mortality

To determine the relations between vaccination status and mortality with other covariates statistically controlled, we applied generalized linear mixed models (GLMMs) nesting COVID-19 patient (*j*) within health system (*i*), with patient-level mortality (*Y*_*ij*_=0 implying no death; 1=death) as the outcome. Patient-level predictors (*X*) included COVID vaccination status, which was coded either as binary (*X*_*ij*1_=0 implying no vaccination; 1 = any vaccination) or by the number of vaccine doses (i.e., continuous: *X*_*ij*1_=0, 1, 2, or 3 doses), with the 7 patient covariates, with product variables between vaccination status and each of the patient covariates. Health system effects were reflected by a system-specific random intercept and a random slope for the vaccine effect. The resulting GLMM can be written as:

$$ logit\Pr \left({Y}_{ij}=1\right)={\beta}_0+\sum \limits_{k=1}^K{X}_{ij k}{\beta}_k+\sum \limits_{k=2}^K{X}_{ij1}{X}_{ij k}{\beta}_{K+k-1}+{b}_{i0}+{b}_{i1}{X}_{ij1} $$

where *β*_0_ denotes a fixed intercept, *β*_*k*_ (k=1, .. K) denote fixed main effects associated with vaccination and other patient characteristics, *β*_*k*_ (*k*=*K*+1, …2*K*−1) denote fixed interaction effects of vaccination with all other patient characteristics, and *b*_*i*0_ and *b*_*i*1_ represent the system-specific random intercept and system-specific random vaccination slope which are assumed to follow a bivariate normal distribution with bivariate mean 0 and covariance matrix Τ. As four of the patient covariates were categorical, multivariate *χ*^2^ tests were applied to separately evaluate the overall main effect and vaccine interaction effects of each categorical covariate. Results were consistent with the lme4 R package (https://cran.r-project.org/web/packages/lme4/index.html) and HLM8 software^15^; we report results from the HLM8, which uses a first-order penalized quasi-likelihood estimator.

A similar GLMM model was run that examined mortality rates as a function of time-since-last-vaccination (see S Methods).

To control for the correlates of vaccination status, we also report logistic regression-based adjustments of the mortality rate estimates accounting for differences in the multivariable distribution of the 7 patient covariates between vaccinated and unvaccinated patients. The adjusted proportions represent the expected mortality rates if the vaccinated and unvaccinated patients shared the same covariate distribution, here defined by the pooled covariate distribution of the vaccinated and unvaccinated patients within month. An additional analysis using covariate adjustment tested an interaction between number of vaccination doses (0–3) and whether or not the patient had an ICD-10 immunocompromised or immunosuppressed condition. This led to determination of mortality rates in a subpopulation from which patients with such diagnoses were removed.

#### Vaccination Associations in Early and Late Study Periods

To further evaluate changes over time in mortality, we dichotomized the full January 2021–January 2022 sample into two subsamples, one comprising patients admitted in the first 7 months (January 1, 2021–July 31, 2021) of the study period and the other sample comprising patients admitted in the remaining 6 months (August 1, 2021–Jan. 31, 2022). We then fit models in which vaccine status predicted mortality, including time interval effects and a vaccine-interval interaction. Models were run with site random effects for the intercept only and for all four effects (intercept, vaccine, interval, and vaccine × interval). These models were run both without and with adjustment for the covariates using the binary vaccination variable. Changes in overall mortality were also analyzed against the patient-level covariates: age, sex, race, ethnicity, BMI, insurance status, and Elixhauser Comorbidity Index score with vaccination history used as a covariate in focused analyses.

Statistical adjustment via logistic regression-based models accounted for differences in the multivariable distribution of patient covariates across groups. The Benjamini-Hochberg (1995) procedure^16^ was applied to control the false discovery rate in multivariable analyses and results of such correction are shown on relevant tables.

## RESULTS

### The Analysis Sample

Overall, 75.2% (*n* = 65,192) of the sample had both a positive PCR test result and a COVID ICD-10 diagnosis, 5.4% (*n* = 4,706) had only a positive PCR test, and 19.4% (*n* = 16,834) had only a COVID ICD-10 diagnosis at the time of hospitalization. Variation in diagnostic indicators may have been due to prioritization of medical intervention and some clinicians’ sense that multiple indicators or COVID-19 were not necessary. A total of 22,792 patients (26%) had received one or more COVID-19 vaccinations (Table 1).

### Participants per Health System

The number of analysis sample patients from each of the 21 participating health systems ranged from 263 to 11,652 (mean=4130 patients, SD=3352; median=2817). Patients had a mean of 10.32 (SD = 17.79) encounters with the healthcare system prior to the index hospitalization.

### Characteristics of the Sample and Vaccination Rates

The characteristics of the patients across the 21 health systems are depicted in Table 1.

### Vaccination Status of the Sample

S Figure 2 presents the rates at which patients were vaccinated across the 12 months of the study period using both the binary and the continuous vaccination variables. Less than half the sample had received any vaccination by the final month and only about 10% had received a 3rd dose by that time. Patterns of vaccine uptake (observed, not covariate adjusted) over time for the binary vaccination variable are depicted for the different covariate populations in S Figures 3–9.

Analyses of rates of vaccination (binary) as a function of covariate population and time were conducted via simultaneous entry of all covariates (S Table 2). There was a significant main effect reflecting overall increases in vaccination rates over time. Groups especially likely to be vaccinated were patients over 60 years of age, males, those on Medicare, and those with higher comorbidity scores (see S Figures 3–9). Groups especially unlikely to be vaccinated were Black patients, Hispanic patients, the severely obese, and those receiving Medicaid or who had missing or other insurance status. Interaction tests between time and covariate groups showed that some groups exhibited especially large increases in vaccination receipt over time, e.g., Black, Asian, and Hispanic individuals (see S Figures 3–9).

### Mortality

The covariate-adjusted mortality rates were 5.1% and 8.3% for vaccinated and unvaccinated patients hospitalized with COVID-19, respectively, in the whole analysis sample; mortality rates were 6.2% and 7.7%, respectively, in unadjusted analyses (Table 2). The adjusted proportions in Table 2 represent the expected mortality rates across categories if the different vaccination groups (either binary or continuous) shared the same pooled covariate distribution. When mortality rates were examined in the sample from which immunocompromised and immunosuppressed patients had been removed (the “Restricted” sample in Table 2), the effect of increasing number of vaccination doses was especially pronounced. Consistent with this, a test of an interaction between immune compromised/suppressed status and number of vaccine doses (0–3) yielded a modest per dose OR = 1.11 (95% CI = 1.02, 1.21: *B* = 0.11) indicating relatively reduced vaccine efficacy in immune compromised/suppressed patients. Analyses showed highly similar relations between dose number and mortality for the Pfizer and Moderna vaccines (data not shown).

**Table 2:** Unadjusted and Adjusted Mortality Rates as a Function of Binary and Categorical Vaccination Status Among COVID-19 Patients Hospitalized from January 2021 to January 2022 with Data Presented for the Whole Analysis Sample and for a Sample from Which Immunocompromised and Immunosuppressed Patients Were Removed (“Restricted”)

Vaccination categorization	Status	*N*	Observed mortality Rate (95% CI)	Adjusted mortality Rate (95% CI)
Full sample		86,732		
Binary	Unvaccinated	63,940	.077 (.075, .079)	.083 (.081, .085)
	Vaccinated	22,792	.062 (.059, .065)	.051 (.048, .054)
Categorical	0 doses	63,940	.077 (.075, .079)	.083 (.081, .085)
	1 dose	5569	.058 (.052, .064)	.053 (.047, .059)
	2 doses	13,647	.064 (.060, .068)	.052 (.048, .055)
	3 doses	3576	.060 (.052, .068)	.046 (.038, .052)
Restricted sample		79,036		
Binary	Unvaccinated	59,703	.075 (.072, .077)	.079 (.076, .082)
	Vaccinated	19,333	.055 (.052, .059)	.045 (.041, .048)
Categorical	0 doses	59,703	.075 (.072, .077)	.079 (.076, .082)
	1 dose	4948	.053 (.047, .059)	.047 (.041, .053)
	2 doses	11,746	.058 (.054, .063)	.047 (.042, .052)
	3 doses	2639	.047 (.039, .056)	.035 (.026, .045)

#### Binary Index

Table 3 presents results relating simultaneously entered covariate categories and binary vaccination status with regard to in-hospital mortality. Receiving any vaccination was associated with reduced mortality (OR = 0.71, CI = 0.53 to 0.94). This association was somewhat larger when immunocompromised and immunosuppressed patients were removed from the sample (OR = 0.62, CI= 0.44 to 0.87: Table 2 and S Table 3). The following variables were significantly related to greater likelihood of mortality in the full sample analysis (Table 3): older age; higher scores on the comorbidity index; male sex; American Indian and Asian races or identifying as more than one race; uninsured health insurance status; and being overweight, obese, or severely obese. Some missing/not reported categories were also significantly positively related to mortality likelihood.

**Table 3:** In-hospital Mortality Predicted from Binary Vaccination Status and Patient Covariates

Predictor	OR	95% CI	*p*
Age	1.04	(1.04, 1.05)	< .001*
Comorbidity Index	1.02	(1.015, 1.021)	< .001*
Sex			
Female (ref)	1.00	--	
Male	1.57	(1.48, 1.67)	< .001*
Race			
White (ref)	1.00	--	
American Indian or Alaska Native	2.22	(1.50, 3.28)	< .001*
Asian	1.93	(1.62, 2.31)	< .001*
Black or African American	0.97	(0.90, 1.05)	.460
Native Hawaiian or Pacific Islander	1.55	(0.93, 2.58)	.096
Other or not specified	1.13	(0.99, 1.29)	.072
More than one	2.20	(1.43, 3.39)	< .001*
Not reported or missing	1.21	(0.96, 1.52)	.109
Ethnicity			
Not Hispanic or Latino (ref)	1.00	--	--
Hispanic or Latino	1.09	(0.97, 1.23)	.163
Not reported or missing	1.48	(1.20, 1.82)	< .001*
Insurance status			
Commercial (ref)	1.00	--	--
Medicare	1.12	(1.01, 1.25)	.026
Medicaid	0.99	(0.87, 1.14)	.924
Uninsured	1.29	(1.05, 1.58)	.015*
Other or missing	1.03	(0.90, 1.19)	.684
Body mass index			
Healthy weight (ref)	1.00	--	--
Underweight	1.06	(0.88, 1.26)	.544
Overweight	1.32	(1.21, 1.44)	< .001*
Obese	1.73	(1.59, 1.89)	< .001*
Severely obese	2.79	(2.50, 3.12)	< .001*
Missing	1.87	(1.44, 2.44)	< .001*
Vaccination	0.71	(0.53, 0.95)	.022
Interaction terms			
Sex			
Male × vaccine	0.94	(0.83, 1.07)	.371
Race			
American Indian or Alaska Native × vaccine	0.64	(0.25, 1.64)	.354
Asian × vaccine	0.86	(0.59, 1.28)	.461
Black or African American × vaccine	1.34	(1.14, 1.58)	< .001*
Native Hawaiian or Pacific Islander × vaccine	0.91	(0.29, 2.88)	.872
Other or not specified × vaccine	0.83	(0.60, 1.14)	.243
More than one × vaccine	0.81	(0.33, 2.02)	.656
Not reported or missing × vaccine	0.92	(0.51, 1.66)	.776
Ethnicity			
Hispanic or Latino × vaccine	1.19	(0.90, 1.58)	.223
Not reported or missing × vaccine	0.82	(0.50, 1.34)	.427
Insurance status			
Medicare × vaccine	1.21	(0.95, 1.54)	.132
Medicaid × vaccine	1.01	(0.73, 1.42)	.934
Uninsured × vaccine	1.07	(0.59, 1.94)	.830
Other or missing × vaccine	0.92	(0.62, 1.38)	.697
Body mass index			
Underweight × vaccine	1.20	(0.87, 1.66)	.262
Overweight × vaccine	0.84	(0.71, 1.00)	.050
Obese × vaccine	0.67	(0.56, 0.80)	< .001*
Severely obese × vaccine	0.52	(0.41, 0.67)	< .001*
Missing × vaccine	0.96	(0.53, 1.72)	.881
**Age** × vaccine	0.99	(0.980, 0.992)	< .001*
**Comorbidity Index** × vaccine	1.00	(0.996, 1.007)	.521
Time			
Month 1 (ref)	1.00	--	--
Month 2	0.88	(0.79, 0.99)	.036
Month 3	0.89	(0.78, 1.00)	.056
Month 4	0.91	(0.80, 1.04)	.165
Month 5	0.85	(0.70, 1.02)	.085
Month 6	0.82	(0.63, 1.07)	.140
Month 7	1.30	(1.10, 1.54)	.002*
Month 8	1.53	(1.37, 1.72)	< .001*
Month 9	1.59	(1.42, 1.79)	< .001*
Month 10	1.54	(1.35, 1.76)	< .001*
Month 11	1.58	(1.39, 1.80)	< .001*
Month 12	1.28	(1.16, 1.42)	< .001*
Month 13	0.86	(0.78, 0.95)	.002*

*Indicates effect remains significant after Benjamini-Hochberg procedure to control false discovery rate

Significant interactions between the covariates and vaccination status were found. Black race was related to significantly less reduction in mortality compared to White race as a function of vaccine receipt (Table 3). In Table 3, the adjusted proportions represent the expected mortality rates across categories if the different vaccination groups shared the same pooled covariate distribution (excluding the race covariate) as defined within the respective race category. Figure 1 shows that both adjusted and unadjusted mortality rates were higher in unvaccinated White patients than in unvaccinated Black patients. Vaccination appeared to provide especially high levels of protection among the obese and severely obese relative to those of healthy weight and to those of older versus younger ages. Results also show that mortality likelihood was significantly higher in each of months 7–12 versus month 1 (see Table 3, S Figure 10a).

#### Mortality Results with a Continuous Index of Vaccination Status

The continuous index of vaccination status was significantly related to mortality (OR = 0.86, CI = 0.75 to 0.99) with statistical adjustment for covariates (S Table 4). This association was somewhat stronger when immunocompromised and immunosuppressed patients were removed from the sample (OR = 0.81, CI= 0.68 to 0.96: S Table 5). The relations of covariates with mortality were relatively unaffected by the use of a continuous index of vaccination status versus the binary vaccine variable (S Table 4). A model was also run with time-since-vaccination entered as a covariate along with a continuous index of vaccination status. This model showed significant but modest effects of time since vaccination (S Methods: Time Since Vaccination).

#### Vaccination Associations in Early and Late Study Periods

The interaction between vaccination status (binary) and time was analyzed with time coded as the first 7 months of the study period (January 1, 2021, to July 31, 2021) and the last 6 months of the study period (August 1, 2021, to January 31, 2022). This analysis (S Table 6) revealed that overall mortality rate increased significantly from the early to the later period in both unadjusted (*t* = 3.01, df = 86,708; OR = 1.10, CI = 1.035 to 1.17) and adjusted (*t* = 7.40, df = 86,687, OR = 1.26, CI = 1.18 to 1. 34) analyses (S Table 6). An interaction effect indicated that the mortality rate among vaccinated persons increased more from the early to later periods than it had among unvaccinated persons (see also Table 4). This interaction effect was significant in unadjusted analyses (*t* = 3.063, df = 86708; *p*=0.002; OR = 1.32, CI = 1.01 to 1.57); however, the covariate-adjusted *p*-value was *p* = 0.051 (*t* = 1.95, df =86687, *p* = 0.051, OR = 1.19, CI = .999–1.42) (see S Table 6). Thus, the data revealed higher mortality rates in the late versus early study periods, with some evidence that the early-to-late increase was greater among vaccinated persons. The relationship of the continuous vaccination metric was not examined given the lack of multi-dose vaccination early in the study period.

**Table 4:** Unadjusted Mortality Rates as a Function of Binary Vaccination in Early (January 2021 to July 2021: *N*=32,548 Unvaccinated, 3696 Vaccinated) and Later (August 2021 to January 2022: *N*=31,392 Unvaccinated, 19,096 Vaccinated) Time Intervals

Period	Vaccine status	Estimate	95% CI
January 2021 to July 2021	Unvaccinated	.075	.072, .077
	Vaccinated	.046	.039, .052
August 2021 to January 2022	Unvaccinated	.079	.076, .082
	Vaccinated	.065	.062, .069

## DISCUSSION

In this large, diverse sample of 86,732 patients with COVID-19 hospitalized from January 1, 2021, to January 31, 2022, vaccination for COVID-19 was associated with significantly reduced mortality. Overall, in analyses in which results were adjusted for covariates that are often associated with COVID-19 severity,^17–19^ the mortality rates were 5.1% and 8.3% for vaccinated and unvaccinated patients, respectively. When patients were “restricted” to those who had no immune compromised or suppressed diagnoses, the association between the number of vaccination doses and mortality became somewhat stronger. Thus, in the Restricted subsample, the adjusted mortality rate for patients who were not immunized was 7.9% while it was only 3.5% in those who had received three vaccine doses. This underscores the value of additional vaccine doses or booster vaccinations even among patients sufficiently ill so as to require hospitalization

Results showed that vaccination rates differed significantly across the study period for different patient groups. Patient groups that were especially likely to be vaccinated were those of older age (60 and older), those on Medicare, and those with higher comorbidity scores, groups that were targeted for early vaccination. In contrast, Black and Hispanic individuals tended to have relatively low vaccination rates^9^ over the study period but also showed the greatest rate of increase over time. Patients on Medicaid and severely obese patients also had low rates of vaccination relative to their comparison conditions. Thus, the findings highlight populations that may benefit from targeting in future vaccination efforts, although these data do not reflect population-based vaccination rates. Relatively low vaccination rates have also been reported for younger, Black, and Hispanic individuals in population-based studies.^20,21^

With statistical adjustment for the covariates, mortality was associated with risk factors for COVID-19 severity identified in earlier research,^17–19^ e.g., older age, comorbid conditions, and male sex. Data were also examined for interactions between covariate categories and vaccination status (vaccinated vs. non-vaccinated). In analyses adjusting for multiple covariates, results showed significantly less reduction in vaccination-related mortality in Black patients versus While patients and significantly greater reduction in mortality in older patients and the obese and severely obese. Black and White patients had similar mortality rates when vaccinated, but when unvaccinated, White patients had significantly higher rates. Most previous research suggests that Black individuals tend to benefit from vaccination as much as White individuals, but little research has been done on this issue in hospitalized samples. One study using a patient population from a single large health system suggests fairly equivalent short-term vaccine effectiveness in Black and White patients as measured by infection rates.^22^ An additional study^7^ of US military veterans reported no significant difference between Black and White individuals in the effectiveness of COVID-19 vaccination in reducing likelihood of laboratory-confirmed COVID-19 infection. We conducted a series of analyses to determine if patterns of individual comorbidities or other risk factors might account for this race difference but no strong evidence was found. More research is needed to understand this difference in mortality risk as a function of race and vaccination status.

Many factors may have affected mortality and vaccine effectiveness over time, including changes in COVID-19 variant distribution, differences in populations being vaccinated, and improvements in patient care and management. Analyses examining mortality showed relatively high mortality rates after study month 7. Analyses of mortality from early (January–July 2021) and later (August 2021 to January 2022) time periods showed that mortality rates increased significantly from the first to the second period with some evidence that they did so especially for vaccinated individuals. We conducted analyses (not presented) that failed to show a meaningful relationship between increased mortality and a surge in hospital admissions. In addition, analyses showed that a time-since-vaccination covariate was only modestly related to mortality (S: Time Since Vaccination) suggesting that this factor was not an important determinant of the increase in mortality among vaccinated patients. The second 6-month time period captures a period of relative elevation of delta variant prevalence (late in 2021^23,24^:. Some data suggest a greater mortality risk with that variant relative to other variants that preceded and followed it (e.g., relative to the alpha variant, which preceded it^23,25^ and the omicron variant, which followed it^23^). In addition, some data also suggest a relatively reduced efficacy of COVID-19 vaccination against this variant versus other variants.^23,26,27^ It is also the case that this study did not distinguish between patients hospitalized primarily for COVID-19 versus for other causes (cf.^23^) because we were interested in the likelihood of mortality among all patients diagnosed with COVID-19, not just those hospitalized primarily for COVID-19. The temporal patterns of mortality observed in this study may have changed had analyses been restricted to the latter group of patients. For instance, during the period of delta variant prominence, a greater proportion of patients may have been hospitalized primarily because of COVID-19 versus for other reasons, meaning that mortality was more likely to reflect severe COVID-19 during that time.

Limitations of this work include the fact that EHR data would not reflect vaccination that was not recorded in the health system EHR^9,28^ Also, mortality rates reflect all-cause mortality; some deaths may have occurred for reasons other than COVID-19 infection. Deaths outside of the healthcare systems and that occurred post-discharge were not available. Additionally, data on hospital features and care and staffing patterns at hospitals were unavailable as were data on type of COVID-19 variants infecting patients. We did not control for laboratory tests of COVID or COVID symptoms since we did not want to control disease severity.

In sum, analyses of a large, diverse sample of over 80,000 COVID-19 patients hospitalized from January 1, 2021, to January 31, 2022, in 21 US health systems demonstrated about a 40% decline in in-hospital mortality among all patients who had received any vaccination as compared with unvaccinated patients. Vaccination reduced the likelihood of mortality by more than half among patients who had three vaccine doses and who were not immune compromised or suppressed. Vaccination was associated with especially large reductions in mortality in obese, severely obese, and older patients, encouraging additional efforts to increase vaccination rates in such patient groups. Unfortunately, increased mortality rates were observed among hospitalized patients late in the 1-year study period, especially among vaccinated patients. This increase deserves additional research attention.

## Supplementary Information

ESM 1 (DOCX 2937 kb)

## References

[1] Centers for Disease Control and Prevention. Covid data tracker. 2022. https://covid.cdc.gov/covid-data-tracker/#demographics. Accessed 24 May 2022

[2] Andrews N, Tessier E, Stowe J (2022). Duration of protection against mild and severe disease by covid-19 vaccines. N Engl J Med..

[3] Danza P, Koo TH, Haddix M (2022). SARS-CoV-2 infection and hospitalization among adults aged >/=18 years, by vaccination status, before and during SARS-CoV-2 B.1.1.529 (Omicron) variant predominance - Los Angeles County, California, November 7, 2021-January 8, 2022. MMWR Morb Mortal Wkly Rep..

[4] Modes ME, Directo MP, Melgar M (2022). Clinical characteristics and outcomes among adults hospitalized with laboratory-confirmed SARS-CoV-2 infection during periods of B.1.617.2 (Delta) and B.1.1.529 (Omicron) variant predominance - one hospital, California, July 15-September 23, 2021, and December 21, 2021-January 27, 2022. MMWR Morb Mortal Wkly Rep..

[5] **Voysey M, Clemens SAC, Madhi SA, et al.** Safety and efficacy of the ChAdOx1 nCoV-19 vaccine (AZD1222) against SARS-CoV-2: an interim analysis of four randomised controlled trials in Brazil, South Africa, and the UK. *Lancet*. Dec 8 2020; 10.1016/S0140-6736(20)32661-1 10.1016/S0140-6736(20)32661-1 PMC7723445 33306989

[6] Abhilash KPP, Mathiyalagan P, Krishnaraj VRK (2022). Impact of prior vaccination with Covishield(TM) and Covaxin(R) on mortality among symptomatic COVID-19 patients during the second wave of the pandemic in South India during April and May 2021: a cohort study. Vaccine..

[7] Young-Xu Y, Korves C, Roberts J (2021). Coverage and estimated effectiveness of mRNA COVID-19 vaccines among US veterans. JAMA Netw Open..

[8] **Cohn BA, Cirillo PM, Murphy CC, Krigbaum NY, Wallace AW.** SARS-CoV-2 vaccine protection and deaths among US veterans during 2021. *Science*. Nov 4 2021:eabm0620. 10.1126/science.abm0620 10.1126/science.abm0620 PMC9836205 34735261

[9] Taylor CA, Whitaker M, Anglin O (2022). COVID-19-associated hospitalizations among adults during SARS-CoV-2 Delta and Omicron variant predominance, by race/ethnicity and vaccination status - COVID-NET, 14 states, July 2021-January 2022. MMWR Morb Mortal Wkly Rep..

[10] Tenforde MW, Self WH, Adams K (2021). Association between mRNA vaccination and covid-19 hospitalization and disease severity. JAMA..

[11] Aslam J, Rauf UL Hassan M, Fatima Q (2022). Association of disease severity and death outcome with vaccination status of admitted COVID-19 patients in delta period of SARS-COV-2 in mixed variety of vaccine background. Saudi J Biol Sci..

[12] Tegria. BlueTree is now Tegria. https://www.tegria.com/bluetree-is-now-tegria/. Accessed 7 July 2022

[13] van Walraven C, Austin PC, Jennings A, Quan H, Forster AJ (2009). A modification of the Elixhauser comorbidity measures into a point system for hospital death using administrative data. Med Care..

[14] National Institute of Health. Racial and ethnic categories and definitions for NIH diversity programs and for other reporting. 2015. https://grants.nih.gov/grants/guide/notice-files/NOT-OD-15-089.html#:~:text=The%20revised%20standards%20contain%20five,%22Not%20Hispanic%20or%20Latino.%22. Accessed 7 July 2022

[15] **Raudenbush SW, Congdon RT.** *HLM 8: Hierarchical linear and nonlinear modeling*. Scientific Software International, Inc.; 2021.

[16] Benjamini Y, Hochberg YL (1995). Controlling the false discovery rate: a practical and powerful approach to multiple testing. J R Stat Soc, Series B Methodol..

[17] Bennett TD, Moffitt RA, Hajagos JG (2021). Clinical characterization and prediction of clinical severity of SARS-CoV-2 infection among US adults using data from the US National COVID Cohort Collaborative. JAMA Netw Open..

[18] Finelli L, Gupta V, Petigara T, Yu K, Bauer KA, Puzniak LA (2021). Mortality among US patients hospitalized with SARS-CoV-2 infection in 2020. JAMA Open..

[19] Harrison SL, Fazio-Eynullayeva E, Lane DA, Underhill P, Lip GYH (2020). Comorbidities associated with mortality in 31,461 adults with COVID-19 in the United States: A federated electronic medical record analysis. PLoS Med..

[20] Diesel J, Sterrett N, Dasgupta S (2021). COVID-19 vaccination coverage among adults - United States, December 14, 2020-May 22, 2021. MMWR Morb Mortal Wkly Rep..

[21] Kriss JL, Hung MC, Srivastav A (2022). COVID-19 vaccination coverage, by race and ethnicity - National Immunization Survey Adult COVID Module, United States, December 2020-November 2021. MMWR Morb Mortal Wkly Rep..

[22] **Butt AA, Omer SB, Yan P, Shaikh OS, Mayr FB.** SARS-CoV-2 vaccine effectiveness in a high-risk national population in a real-world setting *Ann Intern Med*. Jul 20 2021;10.7326/M21-1577 10.7326/M21-1577 PMC8381771 34280332

[23] Adjei S, Hong K, Molinari NM (2022). Mortality risk among patients hospitalized primarily for COVID-19 during the omicron and delta variant pandemic periods - United States, April 2020-June 2022. MMWR Morb Mortal Wkly Rep..

[24] Johnson AG, Amin AB, Ali AR (2022). COVID-19 incidence and death rates among unvaccinated and fully vaccinated adults with and without booster doses during periods of delta and omicron variant emergence - 25 U.S. jurisdictions, April 4-December 25, 2021. MMWR Morb Mortal Wkly Rep..

[25] Centers for Disease Control and Prevention. Covid data tracker weekly review. 2022. https://www.cdc.gov/coronavirus/2019-ncov/covid-data/covidview/past-reports/04222022.html. Accessed 17 Nov 2022

[26] Scobie HM, Johnson AG, Suthar AB (2021). Monitoring incidence of COVID-19 cases, hospitalizations, and deaths, by vaccination status - 13 U.S. jurisdictions, April 4-July 17, 2021. MMWR Morb Mortal Wkly Rep..

[27] Centers for Disease Control and Prevention. Rates of COVID-19 cases and deaths by vaccination status. US Department of Health and Human Service, Centers for Disease Control and Prevention; 2021. https://covid.cdc.gov/covid-data-tracker/#rates-by-vaccine-status. Accessed 17 Novmber 2022

[28] Nyberg T, Ferguson NM, Nash SG (2022). Comparative analysis of the risks of hospitalisation and death associated with SARS-CoV-2 omicron (B.1.1.529) and delta (B.1.617.2) variants in England: a cohort study. Lancet..

