# Supplementary material for: The Relationship of COVID-19 Vaccination with Mortality Among 86,732 Hospitalized Patients: Subpopulations, Patient Factors, and Changes over Time
Source: J Gen Intern Med. 2023 Jan 18;38(5):1248–55. doi: 10.1007/s11606-022-08007-0 (PMC9848037; doi:10.1007/s11606-022-08007-0)
Supplement: Supplementary file 1 — (DOCX 2937 kb) [file 11606_2022_8007_MOESM1_ESM.docx]

**Supplementary Material for “The Relationship of COVID-19 Vaccination with Mortality Among 86,732 Hospitalized Patients: Subpopulations, Patient Factors, and Changes Over Time.”**

**SUPPPLEMENTARY METHODS**

**Data elements extracted from the EHR.**

Each data transfer from the 21 healthcare systems included selected EHR information looking back to February 1, 2020, allowing for both the collection of information from new patients and follow-up data from individuals already in the cohort who received additional care at the health system. These retrospective updates were accomplished by assigning each patient in the data set from each health system an enduring cryptographically processed Patient ID based on the SHA256 algorithm, which yielded a 64-character unique and private hash-based message authentication code (HMAC). Secure transfer of data from each of the 21 health systems was accomplished via the transfer of data files to a secure SFTP (secure shell [SSH] File Transfer Protocol) portal located at the UW-Madison CEC-UW Coordinating Center. EHR data extraction code was provided via Github to all participating health systems with versions for Oracle-based and SQL-based EHR systems. Extraction code was further customized by the Information Technology (IT) staff at each of 21 health systems to account for health-system-specific EHR structures, tables, formats, and terminologies. The data extraction code was designed to locate and export data elements in a relatively uniform manner across the 21 health systems. The customization of the extraction code (i.e., with system-specific modifications) yielded partly harmonized data files for transfer to the CEC-UW Coordinating with the same basic set of data files and EHR variables within each data file, thus allowing later processing, additional harmonization, and ultimate merging of the data files across all 21 health systems according to a common set of specifications.

This data extraction process yielded five separate “source” data files from each of the 21 health systems formatted as comma-separated-values (CSV) files that were converted to SPSS data files at the CEC-UW Coordinating Center. Processing of the five source SPSS data files included preparation of variables for later analysis with appropriate formatting and labeling as well as computation of new variables such as body mass index (BMI) from weight and height. Further harmonization and merging of data across the 21 health systems were accomplished via computer programs developed by data management staff at the CEC-UW Coordinating Center.

Data elements (variables) were extracted from the EHR via specialized programming code that created five data files containing groups of related data elements. These data files included patient- and encounter-level information on: 1) sociodemographic and health characteristics; 2) pre- and post-COVID ICD-10 diagnoses; 3) clinical encounter data including treatment site (e.g., inpatient, outpatient), encounter-based ICD-10 diagnoses, mortality, ICU admission, intubation, and other clinical data; 4) selected laboratory test results linked to encounters; and 5) selected medications linked to encounters.

**Weighted Elixhauser Comorbidity Score Calculation.**

Calculation of the Elixhauser Comorbidity Score used ICD-10 diagnoses from the EHR with a 5-year look back pre-COVID-19. The diagnostic groups were identified by using the ICD-10 diagnosis codes listed in Quan et al., "Coding Algorithms for Defining Comorbidities in ICD-9-CM and ICD-10 Administrative Data", Medical Care:43(11), Nov. 2005 p1130-1139. The coding algorithm scans patient diagnostic codes and identifies whether a given ICD-10 code belongs to one or more of 31 different Elixhauser Comorbidity Groups (see Table below). Each of the Comorbidity Groups is coded as a binary variable where a value of 1 indicates the comorbid condition is present and a value of 0 indicates that the comorbid condition is absent. The total Elixhauser Comorbidity Score is a weighted sum of the 31 binary comorbid conditions, with the weights based on van Walraven et al., “A Modification of the Elixhauser Comorbidity Measures into a Point System for Hospital Death using Administrative Data”, Medical Care, 2009, June 1, pp 626-633 (see Table below). The original SAS code for this program was developed by Quan et al at the University of Calgary, Manitoba Centre for Health Policy and modified by CEC-UW data management staff.

| **Calculation of Weighted Elixhauser Comorbidity Score** | | |
| --- | --- | --- |
| **Comorbidity Group** | **ICD-10 codes** | **van Walraven**  **weights** |
| Congestive heart failure | I09.9, I11.0, I13.0, I13.2, I25.5, I42.0, I42.5 - I42.9, I43.x, I50.x, P29.0 | 7 |
| Cardiac arrhythmias | I44.1 - I44.3, I45.6, I45.9, I47.x - I49.x, R00.0, R00.1, R00.8, T82.1, Z45.0, Z95.0 | 5 |
| Valvular disease | A52.0, I05.x - I08.x, I09.1, I09.8, I34.x - I39.x, Q23.0 - Q23.3, Z95.2 - Z95.4 | -1 |
| Pulmonary circulation disorders | I26.x, I27.x, I28.0, I28.8, I28.9 | 4 |
| Peripheral vascular disorders | I70.x, I71.x, I73.1, I73.8, I73.9, I77.1, I79.0, I79.2, K55.1, K55.8, K55.9, Z95.8, Z95.9 | 2 |
| Hypertension, uncomplicated | I10.x | 0 |
| Hypertension, complicated | I11.x - I13.x, I15.x | 0 |
| Paralysis | G04.1, G11.4, G80.1, G80.2, G81.x, G82.x, G83.0 - G83.4, G83.9 | 7 |
| Other neurological disorders | G10.x - G13.x, G20.x - G22.x, G25.4, G25.5, G31.2, G31.8, G31.9, G32.x, G35.x - G37.x, G40.x, G41.x, G93.1, G93.4, R47.0, R56.x | 6 |
| Chronic pulmonary disease | I27.8, I27.9, J40.x - J47.x, J60.x - J67.x, J68.4, J70.1, J70.3 | 3 |
| Diabetes, uncomplicated | E10.0, E10.1, E10.9, E11.0, E11.1, E11.9, E12.0, E12.1, E12.9, E13.0, E13.1, E13.9, E14.0, E14.1, E14.9 | 0 |
| Diabetes, complicated | E10.2 - E10.8, E11.2 - E11.8, E12.2 - E12.8, E13.2 - E13.8, E14.2 - E14.8 | 0 |
| Hypothyroidism | E00.x - E03.x, E89.0 | 0 |
| Renal failure | I12.0, I13.1, N18.x, N19.x, N25.0, Z49.0 - Z49.2, Z94.0, Z99.2 | 5 |
| Liver disease | B18.x, I85.x, I86.4, I98.2, K70.x, K71.1, K71.3 - K71.5, K71.7, K72.x - K74.x, K76.0, K76.2 - K76.9, Z94.4 | 11 |
| Peptic ulcer disease, excluding bleeding | K25.7, K25.9, K26.7, K26.9, K27.7, K27.9, K28.7, K28.9 | 0 |
| AIDS/HIV | B20.x - B22.x, B24.x | 0 |
| Lymphoma | C81.x - C85.x, C88.x, C96.x, C90.0, C90.2 | 9 |
| Metastatic cancer | C77.x - C80.x | 12 |
| Solid tumour without metastasis | C00.x - C26.x, C30.x - C34.x, C37.x - C41.x, C43.x, C45.x - C58.x, C60.x - C76.x, C97.x | 4 |
| Rheumatoid arthritis/collagen vascular diseases | L94.0, L94.1, L94.3, M05.x, M06.x, M08.x, M12.0, M12.3, M30.x, M31.0 - M31.3, M32.x - M35.x, M45.x, M46.1, M46.8, M46.9 | 0 |
| Coagulopathy: | D65 - D68.x, D69.1, D69.3 - D69.6 | 3 |
| Obesity | E66.x | -4 |
| Weight loss | E40.x - E46.x, R63.4, R64 | 6 |
| Fluid and electrolyte disorders | E22.2, E86.x, E87.x | 5 |
| Blood loss anaemia | D50.0 | -2 |
| Deficiency anaemia | D50.8, D50.9, D51.x - D53.x | -2 |
| Alcohol abuse | F10, E52, G62.1, I42.6, K29.2, K70.0, K70.3, K70.9, T51.x, Z50.2, Z71.4, Z72.1 | 0 |
| Drug abuse | F11.x - F16.x, F18.x, F19.x, Z71.5, Z72.2 | -7 |
| Psychoses | F20.x, F22.x - F25.x, F28.x, F29.x, F30.2, F31.2, F31.5 | 0 |
| Depression | F20.4, F31.3 - F31.5, F32.x, F33.x, F34.1, F41.2, F43.2 | -3 |

**S Methods: Time Since Vaccination**

A multilevel model was run that contained the 7 covariates (age, sex, race, ethnicity, BMI, insurance status, and the Elixhauser comorbidity index). Only patients who had received any vaccination were included in this analysis. The generalized linear mixed model (GLMM) nested COVID-19 patient (j) within health system (i), with patient-level mortality ($Y_{ij}$=0 implying no death; 1=death) as the outcome. Patient-level predictors (X) included COVID vaccination status, which was coded by the number of vaccine doses (i.e., continuous: $X_{ij1}$=0, 1, 2 or 3 doses), with the 7 patient covariates. The adjusted rates represent the expected mortality rates if the time-since-vaccination groups shared the same covariate distribution for vaccinated patients. Dummy coding was used to code time-since-vaccination (days since last COVID-19 vaccination prior to hospital admission) based on quartiles of this temporal variable, producing four categories that can be characterized by their median time-since-last vaccination values: Quartile 1: 18 days; Quartile 2: 112 days; Quartile 3: 207 days; and Quartile 4: 281 days. As the Analysis table below shows, time since last dose was significantly related to mortality with both Quartiles 2 and 3 differing from Quartile 1. However, the differences in mortality were quite modest.

| ***Table for time-since-vaccination.*** *In-hospital mortality predicted from time since last dose (quartile), overall number of vaccination doses, and patient covariates, vaccinated patients only.* | | | |
| --- | --- | --- | --- |
| **Predictor** | **OR** | **95 % CI** | ***p*** |
| **Age** | 1.03 | (1.02, 1.03) | < .001 |
| **Comorbidity Index** | 1.02 | (1.01, 1.02) | < .001 |
| **Sex** |  |  |  |
| Female (ref) | 1.00 | -- |  |
| Male | 1.50 | (1.34, 1.67) | < .001 |
| **Race** |  |  |  |
| White (ref) | 1.00 | -- |  |
| American Indian or Alaska Native | 1.50 | (0.64, 3.52) | .350 |
| Asian | 1.60 | (1.12, 2.28) | .009 |
| Black or African American | 1.24 | (1.07, 1.43) | .003 |
| Native Hawaiian or Pacific Islander | 1.32 | (0.47, 3.71) | .601 |
| Other or Not Specified | 0.93 | (0.69, 1.24) | .602 |
| More than One | 1.69 | (0.76, 3.75) | .201 |
| Not Reported or Missing | 1.07 | (0.62, 1.84) | .818 |
| **Ethnicity** |  |  |  |
| Not Hispanic or Latino (ref) | 1.00 | -- | -- |
| Hispanic or Latino | 1.26 | (0.98, 1.63) | .073 |
| Not Reported or Missing | 1.22 | (0.78, 1.91) | .392 |
| **Insurance Status** |  |  |  |
| Commercial (ref) | 1.00 | -- | -- |
| Medicare | 1.36 | (1.09, 1.70) | .007 |
| Medicaid | 0.98 | (0.72, 1.34) | .918 |
| Uninsured | 1.37 | (0.78, 2.40) | .272 |
| Other or Missing | 0.95 | (0.65, 1.39) | .794 |
| **Body Mass Index** |  |  |  |
| Healthy Weight (ref) | 1.00 | -- | -- |
| Underweight | 1.25 | (0.96, 1.63) | .103 |
| Overweight | 1.12 | (0.97, 1.29) | .138 |
| Obese | 1.18 | (1.01, 1.37) | .033 |
| Severely Obese | 1.48 | (1.19, 1.84) | < .001 |
| Missing | 1.74 | (1.03, 2.93) | .039 |
| **Vaccination Doses** | 0.91 | (0.83, 1.00) | .060 |
| **Time Since Latest Dose** |  |  |  |
| Quartile 1 | 1.00 | -- | -- |
| Quartile 2 | 1.33 | (1.12,1.58) | <.001 |
| Quartile 3 | 1.37 | (1.17, 1.61) | <.001 |
| Quartile 4 | 1.22 | (1.04,1.44) | .016 |

| **Table of Mortality Rates as a Function of Time Since Vaccination** | | | | |
| --- | --- | --- | --- | --- |
| **Quartile** | **Total *N*** | ***N* Died** | **Unadjusted**  **Mortality Rate**  **(95% CI)** | **Adjusted**  **Mortality Rate**  **(95% CI)** |
| 1. *Mdn* = 18 days since last dose | 5679 | 279 | 4.9 (4.4, 5.5) | 5.1 (4.6, 5.7) |
| 2. *Mdn* = 112 days since last dose | 5691 | 358 | 6.3  (5.7, 7.0) | 6.4 (5.8, 7.0) |
| 3. *Mdn* = 207 days since last dose | 5720 | 394 | 6.9 (6.3, 7.6) | 6.5 (5.9, 7.1) |
| 4. *Mdn* = 281 days since last dose | 5699 | 382 | 6.7  (6.1, 7.4) | 6.5 (5.9, 7.1) |

**OTHER SUPPLEMENTARY TABLES**

| **S Table 1.** Observed combinations of vaccines received prior to hospitalization | | | | | | |
| --- | --- | --- | --- | --- | --- | --- |
| **Vaccine Doses** | **Vaccine 1 Maker** | **Vaccine 2 Maker** | **Vaccine 3 Maker** | **N** | **% of Dose Group** | **% of All Vaccinated Patients** |
| 1 | Pfizer |  |  | 2,203 | 39.56 | 9.67 |
|  | Janssen |  |  | 1,912 | 34.33 | 8.39 |
|  | Moderna |  |  | 1,425 | 25.59 | 6.25 |
|  | Unspecified |  |  | 27 | 0.48 | 0.12 |
|  | AstraZeneca |  |  | 2 | 0.04 | 0.01 |
|  | ***Total*** |  |  | 5,569 | 100.00 | 24.43 |
| 2 | Pfizer | Pfizer |  | 8,467 | 62.04 | 37.15 |
|  | Moderna | Moderna |  | 4,831 | 35.40 | 21.2 |
|  | Janssen | Janssen |  | 121 | 0.89 | 0.53 |
|  | Janssen | Pfizer |  | 70 | 0.51 | 0.31 |
|  | Janssen | Moderna |  | 52 | 0.38 | 0.23 |
|  | Unspecified | Unspecified |  | 27 | 0.20 | 0.12 |
|  | Moderna | Pfizer |  | 25 | 0.18 | 0.11 |
|  | Pfizer | Moderna |  | 20 | 0.15 | 0.09 |
|  | Pfizer | Janssen |  | 7 | 0.05 | 0.03 |
|  | Unspecified | Moderna |  | 7 | 0.05 | 0.03 |
|  | Unspecified | Pfizer |  | 6 | 0.04 | 0.03 |
|  | Moderna | Janssen |  | 4 | 0.03 | 0.02 |
|  | Moderna | Unspecified |  | 3 | 0.02 | 0.01 |
|  | AstraZeneca | AstraZeneca |  | 2 | 0.01 | 0.01 |
|  | Sinopharm/BIBP | Sinopharm/BIBP |  | 2 | 0.01 | 0.01 |
|  | Novavax | Novavax |  | 1 | 0.01 | 0.004 |
|  | Unspecified | Janssen |  | 1 | 0.01 | 0.004 |
|  | ***Total*** |  |  | 13,647 | 100.00 | 59.41 |
| 3 | Pfizer | Pfizer | Pfizer | 2,228 | 62.30 | 9.78 |
|  | Moderna | Moderna | Moderna | 1,090 | 30.48 | 4.78 |
|  | Moderna | Moderna | Pfizer | 126 | 3.52 | 0.55 |
|  | Pfizer | Pfizer | Moderna | 74 | 2.07 | 0.32 |
|  | Moderna | Pfizer | Pfizer | 7 | 0.20 | 0.03 |
|  | Unspecified | Moderna | Moderna | 5 | 0.14 | 0.02 |
|  | Unspecified | Unspecified | Pfizer | 5 | 0.14 | 0.02 |
|  | Moderna | Pfizer | Moderna | 4 | 0.11 | 0.02 |
|  | Janssen | Janssen | Pfizer | 3 | 0.08 | 0.01 |
|  | Janssen | Pfizer | Pfizer | 3 | 0.08 | 0.01 |
|  | Moderna | Moderna | Unspecified | 3 | 0.08 | 0.01 |
|  | Pfizer | Pfizer | Unspecified | 3 | 0.08 | 0.01 |
|  | Unspecified | Unspecified | Moderna | 3 | 0.08 | 0.01 |
|  | Unspecified | Unspecified | Unspecified | 3 | 0.08 | 0.01 |
|  | Janssen | Janssen | Moderna | 2 | 0.06 | 0.01 |
|  | Moderna | Unspecified | Unspecified | 2 | 0.06 | 0.01 |
|  | Pfizer | Moderna | Moderna | 2 | 0.06 | 0.01 |
|  | Pfizer | Moderna | Pfizer | 2 | 0.06 | 0.01 |
|  | Pfizer | Pfizer | Janssen | 2 | 0.06 | 0.01 |
|  | Pfizer | Unspecified | Pfizer | 2 | 0.06 | 0.01 |
|  | AstraZeneca | AstraZeneca | Pfizer | 1 | 0.03 | 0.004 |
|  | Janssen | Janssen | Janssen | 1 | 0.03 | 0.004 |
|  | Janssen | Moderna | Moderna | 1 | 0.03 | 0.004 |
|  | Moderna | Janssen | Moderna | 1 | 0.03 | 0.004 |
|  | Moderna | Unspecified | Moderna | 1 | 0.03 | 0.004 |
|  | Pfizer | Janssen | Pfizer | 1 | 0.03 | 0.004 |
|  | Unspecified | Pfizer | Pfizer | 1 | 0.03 | 0.004 |
|  | ***Total*** |  |  | 3,576 | 100.00 | 15.69 |

| ***S Table 2.*** *Predictors of binary vaccination as a function of patient covariates and time.* | | | | |
| --- | --- | --- | --- | --- |
| **Predictor** | **OR** | **95 % CI** | | ***p*** |
| **Time** (months from Jan 2021) | 1.26 | 1.24 | 1.28 | < .001* |
| **Age** |  |  |  |  |
| Under 60 years (ref) | 1.00 | -- | -- |  |
| 60-70 years | 1.52 | 1.33 | 1.74 | < .001* |
| Over 70 years | 2.08 | 1.79 | 2.41 | < .001* |
| **Sex** |  |  |  |  |
| Female (ref) | 1.00 | -- | -- |  |
| Male | 1.10 | 1.01 | 1.20 | .022 |
| **Race** |  |  |  |  |
| White (ref) |  |  |  |  |
| American Indian or Alaska Native | 0.80 | 0.36 | 1.59 | .548 |
| Asian | 1.03 | 0.80 | 1.30 | .827 |
| Black or African American | 0.72 | 0.64 | 0.80 | < .001* |
| Native Hawaiian or Pacific Islander | 0.99 | 0.46 | 1.94 | .984 |
| Other or Not Specified | 0.82 | 0.67 | 0.99 | .045 |
| More than One | 0.71 | 0.29 | 1.52 | .414 |
| Not Reported or Missing | 0.90 | 0.61 | 1.30 | .585 |
| **Ethnicity** |  |  |  |  |
| Not Hispanic or Latino (ref) | 1.00 | -- | -- |  |
| Hispanic or Latino | 0.84 | 0.70 | 0.99 | .046 |
| Not Reported or Missing | 1.02 | 0.73 | 1.42 | .887 |
| **Insurance Status** |  |  |  |  |
| Commercial (ref) | 1.00 | -- | -- |  |
| Medicare | 1.31 | 1.14 | 1.52 | < .001* |
| Medicaid | 0.65 | 0.53 | 0.79 | < .001* |
| Uninsured | 0.71 | 0.47 | 1.04 | .094 |
| Other or Missing | 0.68 | 0.54 | 0.86 | .001* |
| **Body Mass Index** |  |  |  |  |
| Healthy Weight (ref) | 1.00 | -- | -- |  |
| Underweight | 0.80 | 0.62 | 1.02 | .080 |
| Overweight | 1.12 | 0.99 | 1.25 | .055 |
| Obese | 1.04 | 0.93 | 1.17 | .457 |
| Severely Obese | 1.26 | 1.08 | 1.48 | .004* |
| Missing | 0.71 | 0.41 | 1.17 | .206 |
| **Comorbidity Index** | 1.04 | 1.04 | 1.04 | < .001* |
| **Interaction Terms** |  |  |  |  |
| **Age** |  |  |  |  |
| 60-70 years × Time | 0.99 | 0.97 | 1.00 | .062 |
| Over 70 years × Time | 0.98 | 0.96 | 0.99 | .004* |
| **Sex** |  |  |  |  |
| Male × Time | 1.00 | 0.99 | 1.00 | .265 |
| **Race** |  |  |  |  |
| American Indian or Alaska Native × Time | 1.02 | 0.95 | 1.11 | .612 |
| Asian × Time | 1.03 | 1.00 | 1.06 | .047 |
| Black or African American × Time | 1.03 | 1.02 | 1.04 | < .001* |
| Native Hawaiian or Pacific Islander × Time | 1.01 | 0.94 | 1.09 | .856 |
| Other or Not Specified × Time | 1.03 | 1.00 | 1.05 | .018 |
| More than One × Time | 1.04 | 0.96 | 1.13 | .381 |
| Not Reported or Missing × Time | 0.99 | 0.95 | 1.03 | .627 |
| **Ethnicity** |  |  |  |  |
| Hispanic or Latino × Time | 1.03 | 1.01 | 1.04 | .008* |
| Not Reported or Missing × Time | 0.99 | 0.96 | 1.03 | .652 |
| **Body Mass Index** |  |  |  |  |
| Underweight × Time | 0.99 | 0.97 | 1.00 | .155 |
| Overweight × Time | 1.01 | 0.99 | 1.03 | .563 |
| Obese × Time | 0.97 | 0.93 | 1.01 | .087 |
| Severely Obese × Time | 0.98 | 0.96 | 1.01 | .182 |
| Missing × Time | 1.01 | 0.98 | 1.03 | .640 |
| **Insurance Status** |  |  |  |  |
| Medicare × Time | 0.99 | 0.98 | 1.01 | .265 |
| Medicaid × Time | 1.00 | 0.99 | 1.01 | .999 |
| Uninsured × Time | 0.98 | 0.96 | 0.99 | .012* |
| Other or Missing × Time | 1.01 | 0.96 | 1.07 | .723 |
| *Indicates effect remains significant after Benjamini-Hochberg procedure to control false discovery rate. | | | | |

| ***S Table 3.*** *In-hospital mortality predicted from binary vaccination status and patient covariates with immunocompromised and suppressed patients removed.* | | | |
| --- | --- | --- | --- |
| **Predictor** | **OR** | **95 % CI** | ***p*** |
| **Age** | 1.04 | (1.04, 1.05) | < .001 |
| **Comorbidity Index** | 1.02 | (1.012, 1.019) | < .001 |
| **Sex** |  |  |  |
| Female (ref) | 1.00 | -- |  |
| Male | 1.60 | (1.50, 1.70) | < .001 |
| **Race** |  |  |  |
| White (ref) | 1.00 | -- |  |
| American Indian or Alaska Native | 2.09 | (1.36, 3.21) | .001 |
| Asian | 1.92 | (1.60, 2.30) | < .001 |
| Black or African American | 0.95 | (0.88, 1.04) | .277 |
| Native Hawaiian or Pacific Islander | 1.68 | (0.99, 2.85) | .056 |
| Other or Not Specified | 1.11 | (0.97, 1.28) | .142 |
| More than One | 2.20 | (1.39, 3.48) | .001 |
| Not Reported or Missing | 1.15 | (0.91, 1.47) | .248 |
| **Ethnicity** |  |  |  |
| Not Hispanic or Latino (ref) | 1.00 | -- | -- |
| Hispanic or Latino | 1.14 | (1.01, 1.30) | .042 |
| Not Reported or Missing | 1.52 | (1.23, 1.89) | < .001 |
| **Insurance Status** |  |  |  |
| Commercial (ref) | 1.00 | -- | -- |
| Medicare | 1.13 | (1.01, 1.26) | .037 |
| Medicaid | 1.02 | (0.88, 1.17) | .826 |
| Uninsured | 1.30 | (1.05, 1.61) | .016 |
| Other or Missing | 1.06 | (0.92, 1.23) | .418 |
| **Body Mass Index** |  |  |  |
| Healthy Weight (ref) | 1.00 | -- | -- |
| Underweight | 1.09 | (0.90, 1.31) | .377 |
| Overweight | 1.31 | (1.19, 1.44) | < .001 |
| Obese | 1.74 | (1.59, 1.90) | < .001 |
| Severely Obese | 2.88 | (2.56, 3.23) | < .001 |
| Missing | 1.91 | (1.46, 2.51) | < .001 |
| **Vaccination** | 0.62 | (0.44, 0.87) | .009 |
| **Interaction Terms** |  |  |  |
| **Sex** |  |  |  |
| Male × Vaccine | 0.94 | (0.82, 1.09) | .432 |
| **Race** |  |  |  |
| American Indian or Alaska Native × Vaccine | 0.33 | (0.08, 1.49) | .150 |
| Asian × Vaccine | 0.83 | (0.53, 1.31) | .425 |
| Black or African American × Vaccine | 1.39 | (1.16, 1.68) | < .001 |
| Native Hawaiian or Pacific Islander × Vaccine | 0.63 | (0.14, 2.92) | .555 |
| Other or Not Specified × Vaccine | 0.84 | (0.59, 1.20) | .338 |
| More than One × Vaccine | 0.74 | (0.24, 2.31) | .605 |
| Not Reported or Missing × Vaccine | 1.12 | (0.59, 2.11) | .733 |
| **Ethnicity** |  |  |  |
| Hispanic or Latino × Vaccine | 1.15 | (0.83, 1.58) | .401 |
| Not Reported or Missing × Vaccine | 0.74 | (0.42, 1.30) | .299 |
| **Insurance Status** |  |  |  |
| Medicare × Vaccine | 1.19 | (0.88, 1.60) | .263 |
| Medicaid × Vaccine | 1.06 | (0.72, 1.58) | .756 |
| Uninsured × Vaccine | 1.05 | (0.53, 2.07) | .886 |
| Other or Missing × Vaccine | 0.93 | (0.58, 1.49) | .765 |
| **Body Mass Index** |  |  |  |
| Underweight × Vaccine | 1.22 | (0.86, 1.73) | .260 |
| Overweight × Vaccine | 0.82 | (0.68, 1.00) | .045 |
| Obese × Vaccine | 0.63 | (0.52, 0.77) | < .001 |
| Severely Obese × Vaccine | 0.53 | (0.40, 0.70) | < .001 |
| Missing × Vaccine | 1.02 | (0.55, 1.87) | .957 |
| **Age** × Vaccine | 0.99 | (0.983,0.997) | .006 |
| **Comorbidity Index** × Vaccine | 1.00 | (0.996,1.008) | .509 |
| **Time** |  |  |  |
| Month 1 (ref) | 1.00 | -- | -- |
| Month 2 | 0.88 | (0.78, 0.99) | .039 |
| Month 3 | 0.89 | (0.78, 1.01) | .076 |
| Month 4 | 0.89 | (0.77, 1.03) | .107 |
| Month 5 | 0.81 | (0.66, 1.00) | .045 |
| Month 6 | 0.78 | (0.59, 1.05) | .102 |
| Month 7 | 1.26 | (1.05, 1.51) | .013 |
| Month 8 | 1.54 | (1.36, 1.74) | < .001 |
| Month 9 | 1.58 | (1.40, 1.79) | < .001 |
| Month 10 | 1.54 | (1.34, 1.78) | < .001 |
| Month 11 | 1.56 | (1.36, 1.79) | < .001 |
| Month 12 | 1.26 | (1.13, 1.40) | < .001 |
| Month 13 | 0.90 | (0.81, 0.99) | .034 |

| ***S Table 4.***  *Mortality predicted from continuous vaccine* | | | |
| --- | --- | --- | --- |
| **Predictor** | **OR** | **95 % CI** | ***p*** |
| **Age** | 1.04 | (1.04, 1.05) | < .001 |
| **Comorbidity Index** | 1.02 | (1.01, 1.02) | < .001 |
| **Sex** |  |  |  |
| Female (ref) | 1.00 | -- |  |
| Male | 1.56 | (1.47, 1.66) | < .001 |
| **Race** |  |  |  |
| White (ref) | 1.00 | -- |  |
| American Indian or Alaska Native | 2.08 | (1.40, 3.08) | < .001 |
| Asian | 1.91 | (1.60, 2.27) | < .001 |
| Black or African American | 0.98 | (0.91, 1.06) | .660 |
| Native Hawaiian or Pacific Islander | 1.36 | (0.80, 2.31) | .258 |
| Other or Not Specified | 1.12 | (0.98, 1.28) | .099 |
| More than One | 2.33 | (1.53, 3.55) | < .001 |
| Not Reported or Missing | 1.23 | (0.98, 1.54) | .078 |
| **Ethnicity** |  |  |  |
| Not Hispanic or Latino (ref) | 1.00 | -- | -- |
| Hispanic or Latino | 1.11 | (0.98, 1.25) | .098 |
| Not Reported or Missing | 1.49 | (1.21, 1.83) | < .001 |
| **Insurance Status** |  |  |  |
| Commercial (ref) | 1.00 | -- | -- |
| Medicare | 1.14 | (1.03, 1.26) | .013 |
| Medicaid | 1.00 | (0.87, 1.14) | .967 |
| Uninsured | 1.30 | (1.06, 1.59) | .013 |
| Other or Missing | 1.04 | (0.90, 1.19) | .603 |
| **Body Mass Index** |  |  |  |
| Healthy Weight (ref) | 1.00 | -- | -- |
| Underweight | 1.05 | (0.88, 1.25) | .612 |
| Overweight | 1.32 | (1.21, 1.44) | < .001 |
| Obese | 1.72 | (1.58, 1.88) | < .001 |
| Severely Obese | 2.76 | (2.48, 3.08) | < .001 |
| Missing | 1.91 | (1.47, 2.48) | < .001 |
| **Vaccination** | 0.86 | (0.75, 0.99) | .042 |
| **Interaction Terms** |  |  |  |
| **Sex** |  |  |  |
| Male × Vaccine | 0.97 | (0.91, 1.04) | .432 |
| **Race** |  |  |  |
| American Indian or Alaska Native × Vaccine | 0.95 | (0.63, 1.44) | .806 |
| Asian × Vaccine | 0.97 | (0.81, 1.17) | .748 |
| Black or African American × Vaccine | 1.13 | (1.04, 1.22) | .005 |
| Native Hawaiian or Pacific Islander × Vaccine | 1.25 | (0.78, 2.01) | .355 |
| Other or Not Specified × Vaccine | 0.93 | (0.79, 1.09) | .354 |
| More than One × Vaccine | 0.79 | (0.49, 1.28) | .343 |
| Not Reported or Missing × Vaccine | 0.88 | (0.64, 1.23) | .463 |
| **Ethnicity** |  |  |  |
| Hispanic or Latino × Vaccine | 1.06 | (0.92, 1.23) | .423 |
| Not Reported or Missing × Vaccine | 0.88 | (0.68, 1.14) | .333 |
| **Insurance Status** |  |  |  |
| Medicare × Vaccine | 1.06 | (0.94, 1.20) | .352 |
| Medicaid × Vaccine | 0.98 | (0.82, 1.17) | .817 |
| Uninsured × Vaccine | 1.01 | (0.74, 1.38) | .950 |
| Other or Missing × Vaccine | 0.92 | (0.74, 1.14) | .458 |
| **Body Mass Index** |  |  |  |
| Underweight × Vaccine | 1.12 | (0.96, 1.30) | .164 |
| Overweight × Vaccine | 0.92 | (0.85, 1.00) | .050 |
| Obese × Vaccine | 0.82 | (0.76, 0.90) | < .001 |
| Severely Obese × Vaccine | 0.71 | (0.63, 0.81) | < .001 |
| Missing × Vaccine | 0.93 | (0.69, 1.24) | .611 |
| **Age** × Vaccine | 0.99 | (0.991, .997) | < .001 |
| **Comorbidity Index** × Vaccine | 1.00 | (0.999, 1.004) | .302 |
| **Time** |  |  |  |
| Month 1 (ref) | 1.00 | -- | -- |
| Month 2 | 0.87 | (0.77, 0.98) | .020 |
| Month 3 | 0.87 | (0.77, 0.98) | .024 |
| Month 4 | 0.89 | (0.78, 1.02) | .091 |
| Month 5 | 0.83 | (0.69, 1.01) | .057 |
| Month 6 | 0.80 | (0.62, 1.05) | .106 |
| Month 7 | 1.28 | (1.08, 1.52) | .004 |
| Month 8 | 1.51 | (1.34, 1.69) | < .001 |
| Month 9 | 1.56 | (1.39, 1.75) | < .001 |
| Month 10 | 1.51 | (1.33, 1.73) | < .001 |
| Month 11 | 1.56 | (1.37, 1.77) | < .001 |
| Month 12 | 1.28 | (1.15, 1.41) | < .001 |
| Month 13 | 0.86 | (0.79, 0.95) | .003 |

| ***S Table 5.***  *Mortality predicted from continuous vaccine with immunocompromised and immunosuppressed patients excluded* | | | |
| --- | --- | --- | --- |
| **Predictor** | **OR** | **95 % CI** | ***p*** |
| **Age** | 1.04 | (1.04, 1.05) | < .001 |
| **Comorbidity Index** | 1.01 | (1.01, 1.02) | < .001 |
| **Sex** |  |  |  |
| Female (ref) | 1.00 | -- |  |
| Male | 1.59 | (1.50, 1.70) | < .001 |
| **Race** |  |  |  |
| White (ref) | 1.00 | -- |  |
| American Indian or Alaska Native | 2.05 | (1.33, 3.16) | .001 |
| Asian | 1.88 | (1.56, 2.25) | < .001 |
| Black or African American | 0.97 | (0.89, 1.05) | .439 |
| Native Hawaiian or Pacific Islander | 1.56 | (0.91, 2.68) | .107 |
| Other or Not Specified | 1.10 | (0.96, 1.26) | .185 |
| More than One | 2.23 | (1.42, 3.52) | < .001 |
| Not Reported or Missing | 1.16 | (0.91, 1.47) | .232 |
| **Ethnicity** |  |  |  |
| Not Hispanic or Latino (ref) | 1.00 | -- | -- |
| Hispanic or Latino | 1.16 | (1.02, 1.31) | .024 |
| Not Reported or Missing | 1.52 | (1.23, 1.88) | < .001 |
| **Insurance Status** |  |  |  |
| Commercial (ref) | 1.00 | -- | -- |
| Medicare | 1.14 | (1.02, 1.27) | .021 |
| Medicaid | 1.02 | (0.89, 1.18) | .733 |
| Uninsured | 1.31 | (1.06, 1.62) | .012 |
| Other or Missing | 1.08 | (0.93, 1.25) | .301 |
| **Body Mass Index** |  |  |  |
| Healthy Weight (ref) | 1.00 | -- | -- |
| Underweight | 1.08 | (0.90, 1.30) | .388 |
| Overweight | 1.31 | (1.19, 1.44) | < .001 |
| Obese | 1.73 | (1.58, 1.90) | < .001 |
| Severely Obese | 2.88 | (2.57, 3.23) | < .001 |
| Missing | 1.95 | (1.50, 2.55) | < .001 |
| **Vaccination** | 0.81 | (0.68, 0.96) | .020 |
| **Interaction Terms** |  |  |  |
| **Sex** |  |  |  |
| Male × Vaccine | 0.97 | (0.90, 1.05) | .424 |
| **Race** |  |  |  |
| American Indian or Alaska Native × Vaccine | 0.59 | (0.27, 1.30) | .192 |
| Asian × Vaccine | 0.98 | (0.79, 1.21) | .864 |
| Black or African American × Vaccine | 1.14 | (1.04, 1.26) | .007 |
| Native Hawaiian or Pacific Islander × Vaccine | 1.02 | (0.55, 1.92) | .942 |
| Other or Not Specified × Vaccine | 0.93 | (0.77, 1.13) | .488 |
| More than One × Vaccine | 0.83 | (0.46, 1.50) | .532 |
| Not Reported or Missing × Vaccine | 1.03 | (0.73, 1.46) | .857 |
| **Ethnicity** |  |  |  |
| Hispanic or Latino × Vaccine | 1.03 | (0.87, 1.22) | .718 |
| Not Reported or Missing × Vaccine | 0.85 | (0.63, 1.14) | .282 |
| **Insurance Status** |  |  |  |
| Medicare × Vaccine | 1.04 | (0.88, 1.22) | .667 |
| Medicaid × Vaccine | 0.97 | (0.78, 1.21) | .766 |
| Uninsured × Vaccine | 0.96 | (0.66, 1.40) | .823 |
| Other or Missing × Vaccine | 0.86 | (0.66, 1.13) | .279 |
| **Body Mass Index** |  |  |  |
| Underweight × Vaccine | 1.11 | (0.94, 1.32) | .215 |
| Overweight × Vaccine | 0.90 | (0.82, 0.99) | .030 |
| Obese × Vaccine | 0.79 | (0.72, 0.87) | < .001 |
| Severely Obese × Vaccine | 0.69 | (0.59, 0.80) | < .001 |
| Missing × Vaccine | 0.96 | (0.70, 1.30) | .786 |
| **Age** × Vaccine | 1.00 | (0.99, 1.00) | .044 |
| **Comorbidity Index** × Vaccine | 1.00 | (0.999, 1.006) | .118 |
| **Time** |  |  |  |
| Month 1 (ref) | 1.00 | -- | -- |
| Month 2 | 0.87 | (0.77, 0.98) | .023 |
| Month 3 | 0.87 | (0.76, 0.99) | .035 |
| Month 4 | 0.87 | (0.75, 1.01) | .059 |
| Month 5 | 0.80 | (0.65, 0.98) | .032 |
| Month 6 | 0.77 | (0.58, 1.03) | .081 |
| Month 7 | 1.24 | (1.04, 1.49) | .020 |
| Month 8 | 1.52 | (1.34, 1.71) | < .001 |
| Month 9 | 1.56 | (1.38, 1.76) | < .001 |
| Month 10 | 1.52 | (1.33, 1.76) | < .001 |
| Month 11 | 1.55 | (1.35, 1.78) | < .001 |
| Month 12 | 1.26 | (1.13, 1.41) | < .001 |
| Month 13 | 0.91 | (0.82, 1.00) | .059 |

| ***S Table 6.*** *In-hospital mortality predicted from binary vaccination status, time interval, and patient covariates.* | | | | | | |
| --- | --- | --- | --- | --- | --- | --- |
|  | ***Random Intercept Only*** | | | ***Random Intercept + Adjustments*** | | |
| **Predictor** | **OR** | **95 % CI** | ***P*** | **OR** | **95 % CI** | ***P*** |
| **Vaccination** | 0.63 | (0.54, 0.74) | <. 001 | 0.47 | (0.40, 0.56) | < .001 |
| **Time Interval** | 1.10 | (1.04, 1.17) | .002 | 1.26 | (1.18, 1.34) | < .001 |
| **Vaccination × Time Interval** | 1.32 | (1.10, 1.57) | .002 | 1.19 | (1.00, 1.42) | .051 |
| **Sex** |  |  |  |  |  |  |
| Female (ref) |  |  |  | 1.00 | -- | -- |
| Male |  |  |  | 1.55 | (1.47, 1.63) | < .001 |
| **Race** |  |  |  |  |  |  |
| White (ref) |  |  |  | 1.00 | -- | -- |
| American Indian or Alaska Native |  |  |  | 2.00 | (1.41, 2.84) | < .001 |
| Asian |  |  |  | 1.82 | (1.56, 2.13) | < .001 |
| Black or African American |  |  |  | 1.00 | (0.94, 1.08) | .892 |
| Native Hawaiian or Pacific Islander |  |  |  | 1.46 | (0.92, 0.92) | .108 |
| Other or Not Specified |  |  |  | 1.08 | (0.96, 1.22) | .225 |
| More than One |  |  |  | 2.04 | (1.39, 2.98) | < .001 |
| Not Reported or Missing |  |  |  | 1.18 | (0.95, 1.46) | .126 |
| **Ethnicity** |  |  |  |  |  |  |
| Not Hispanic or Latino (ref) |  |  |  | 1.00 | -- | -- |
| Hispanic or Latino |  |  |  | 1.11 | (1.00, 1.25) | .054 |
| Not Reported or Missing |  |  |  | 1.41 | (1.17, 1.71) | < .001 |
| **Insurance Status** |  |  |  |  |  |  |
| Commercial (ref) |  |  |  |  |  |  |
| Medicare |  |  |  | 1.15 | (1.05, 1.26) | .003 |
| Medicaid |  |  |  | 0.96 | (0.85, 1.08) | .496 |
| Uninsured |  |  |  | 1.25 | (1.04, 1.52) | .021 |
| Other or Missing |  |  |  | 1.01 | (0.89, 1.15) | .851 |
| **Body Mass Index** |  |  |  |  |  |  |
| Healthy Weight (ref) |  |  |  | 1.00 | -- | -- |
| Underweight |  |  |  | 1.11 | (0.96, 1.29) | .170 |
| Overweight |  |  |  | 1.27 | (1.18, 1.37) | < .001 |
| Obese |  |  |  | 1.59 | (1.48, 1.72) | < .001 |
| Severely Obese |  |  |  | 2.45 | (2.22, 2.7) | < .001 |
| Missing |  |  |  | 1.79 | (1.42, 2.26) | < .001 |
| **Comorbidity Index** |  |  |  | 1.02 | (1.02, 1.02) | < .001 |
| **Age** |  |  |  | 1.04 | (1.04, 1.04) | < .001 |

**SUPPLEMENTARY FIGURES**

**S Figure 1.** Geographic Map of CEC-UW Participating Health System Locations


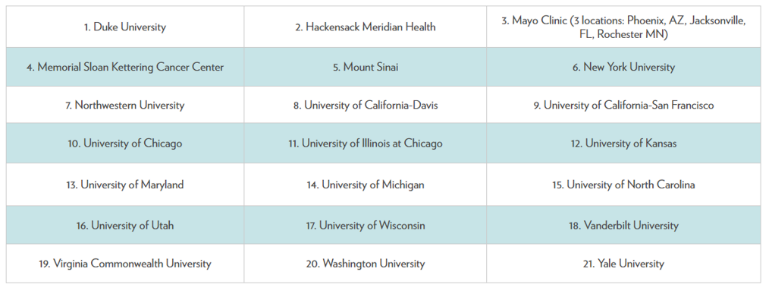

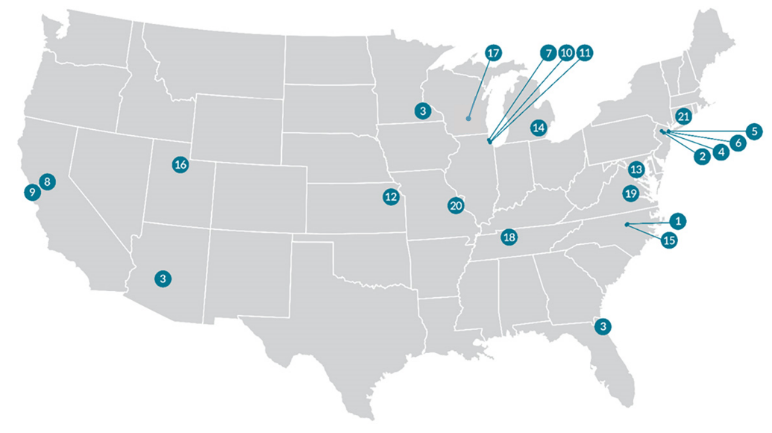

**S Figure 2.** Rates of vaccination across the study period with regard to both the binary (vaccinated and unvaccinated) and the continuous (0, 1, 2, or 3 vaccine doses) across the study period.

**S Figure 3.** Vaccination Rates as a Function of Age


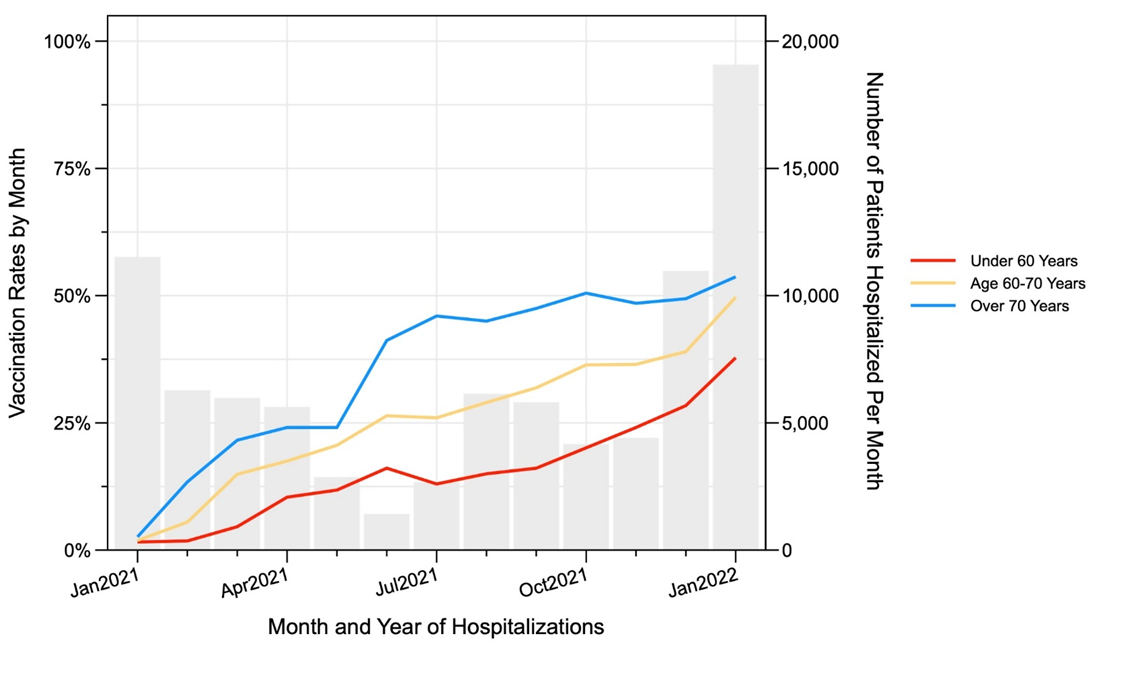


**S Figure 4.** Vaccination Rates as a Function of Sex


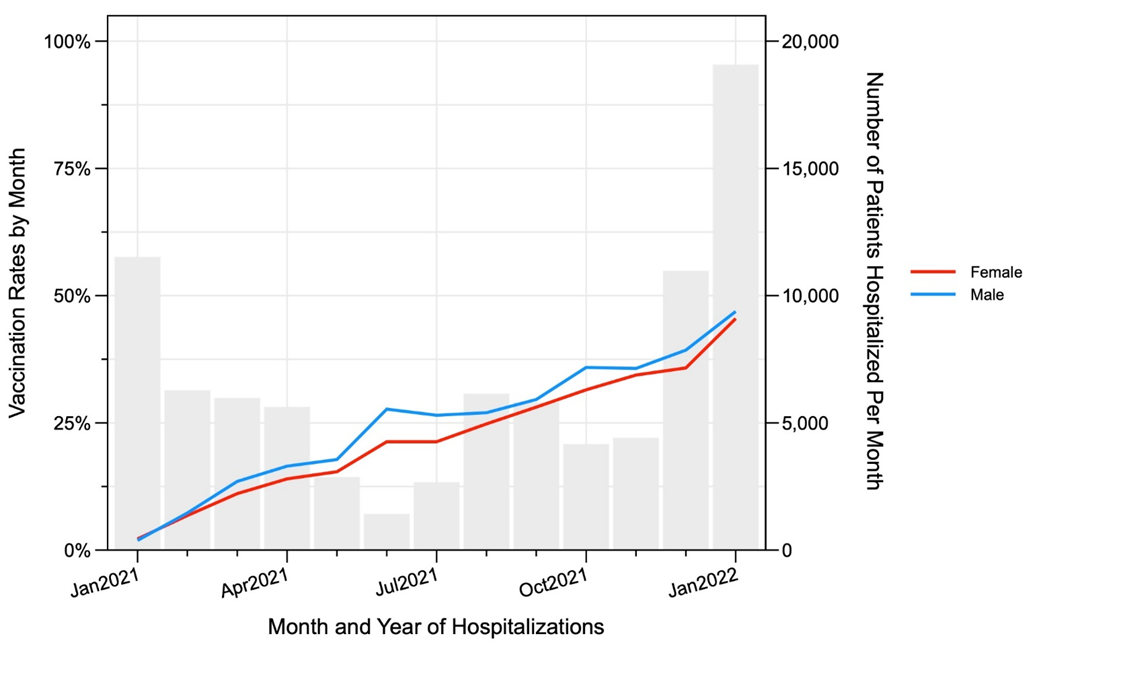


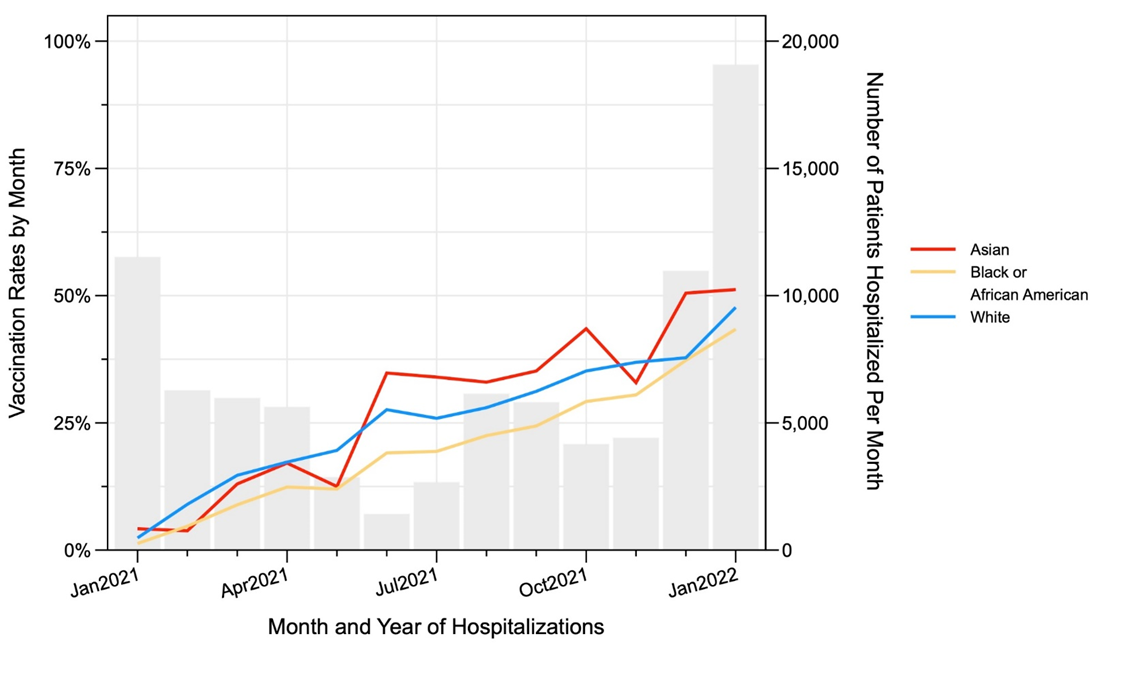
**S Figure 5.** Vaccination Rates as a Function of 3 Race Groups


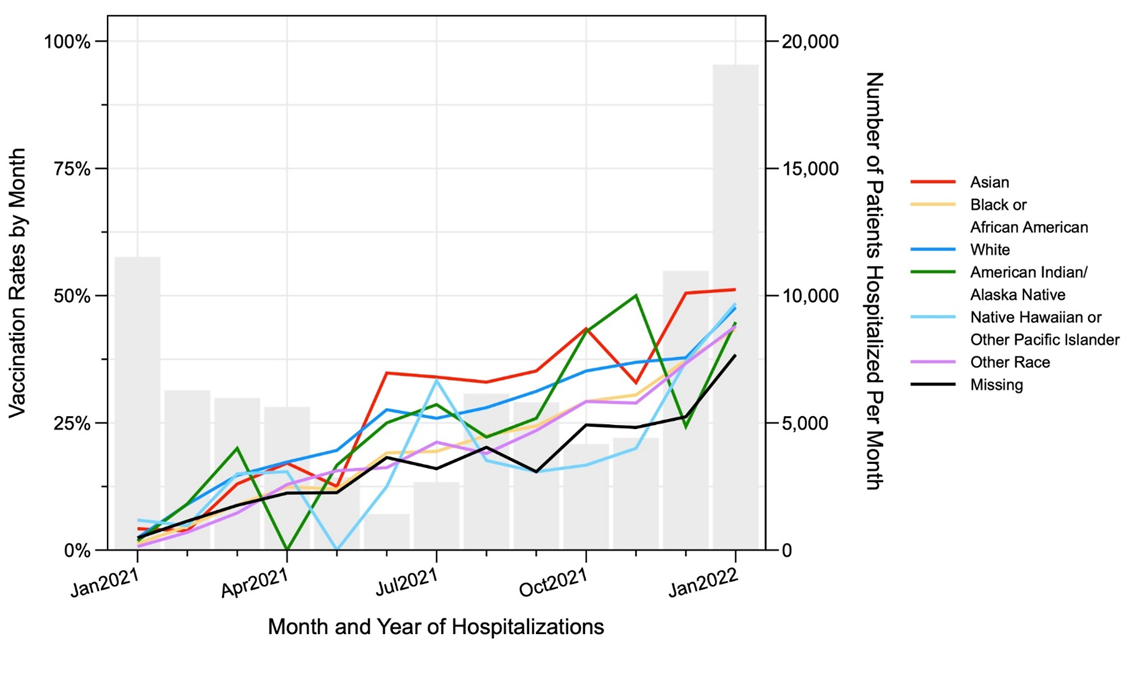
**S Figure 6.** Vaccination Rates as a Function of 7 Race Categories


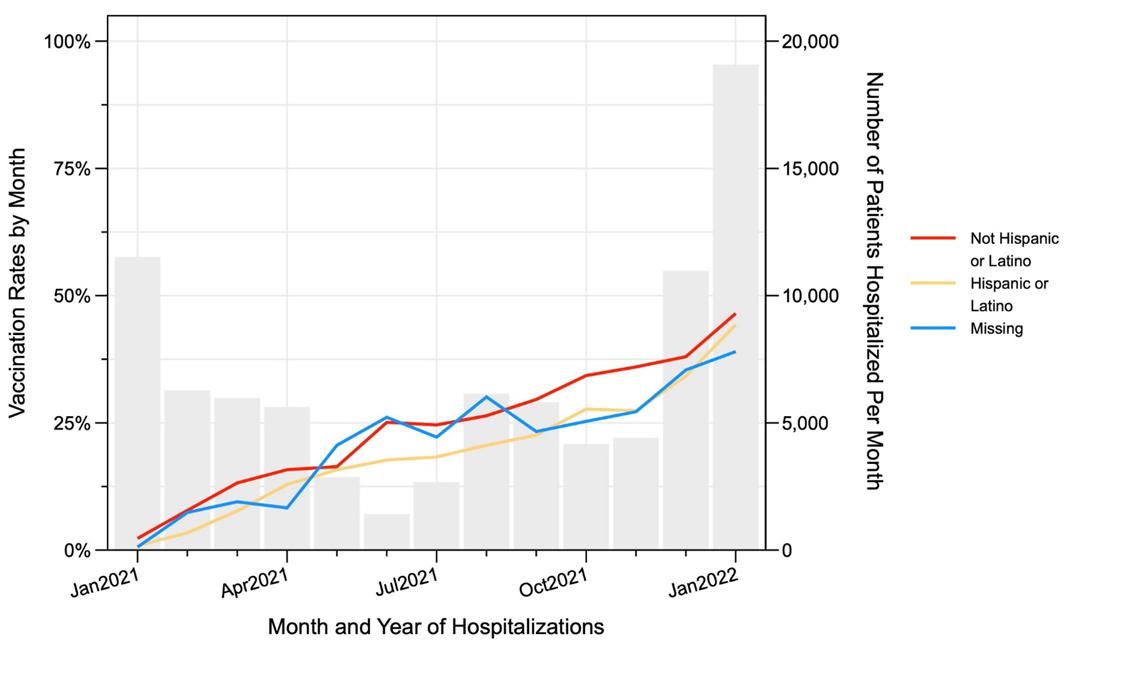
**S Figure 7.** Vaccination Rates as a Function of Ethnicity

**S Figure 8.** Vaccination Rates as a Function of Weight Status


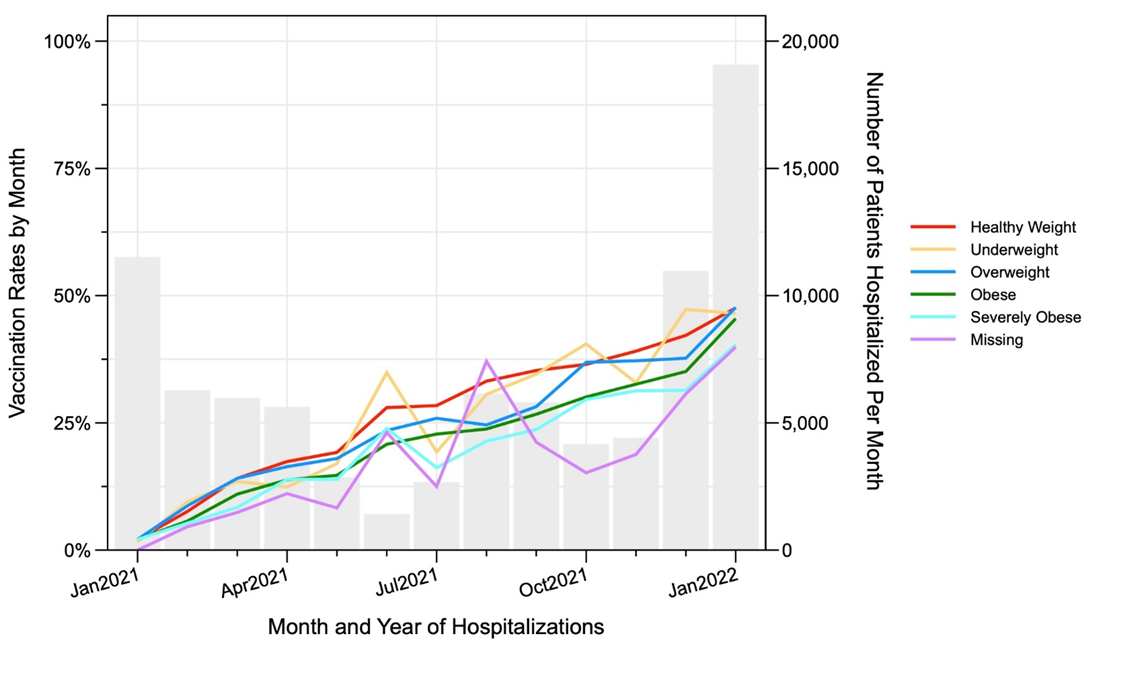


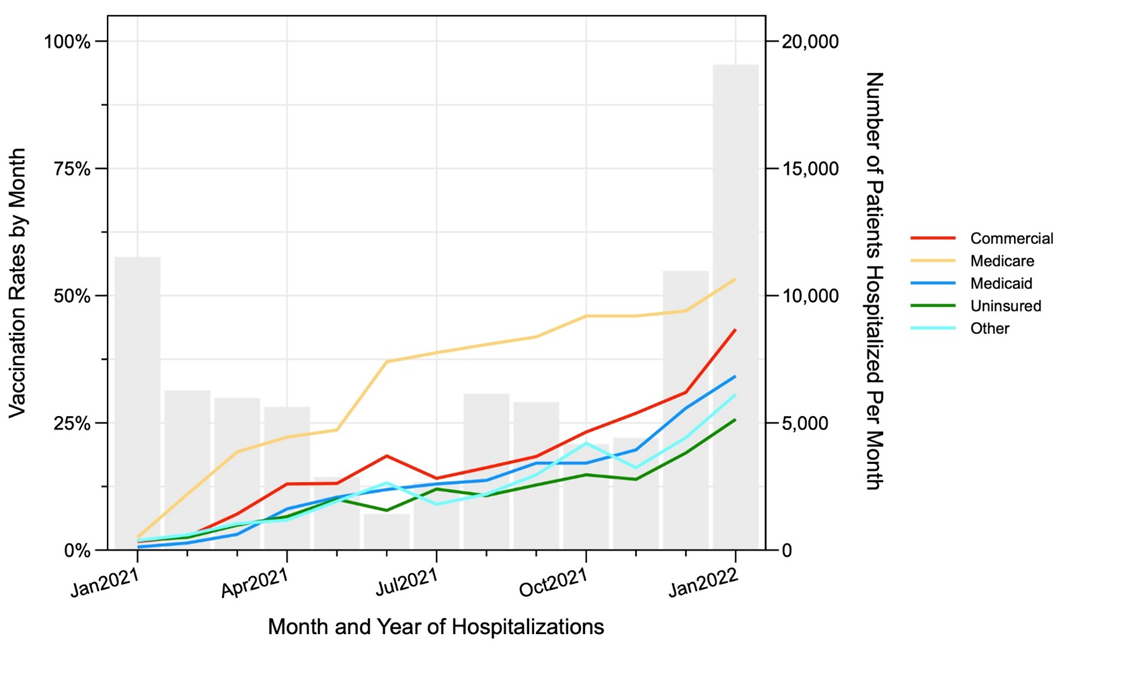
**S Figure 9.** Vaccination Rates as a Function of Insurance Status

**S Figure 10a.** Adjusted and unadjusted mortality rates in vaccinated and unvaccinated patients (the binary measure of vaccination status) over the study period along with rates of patients hospitalized per month.


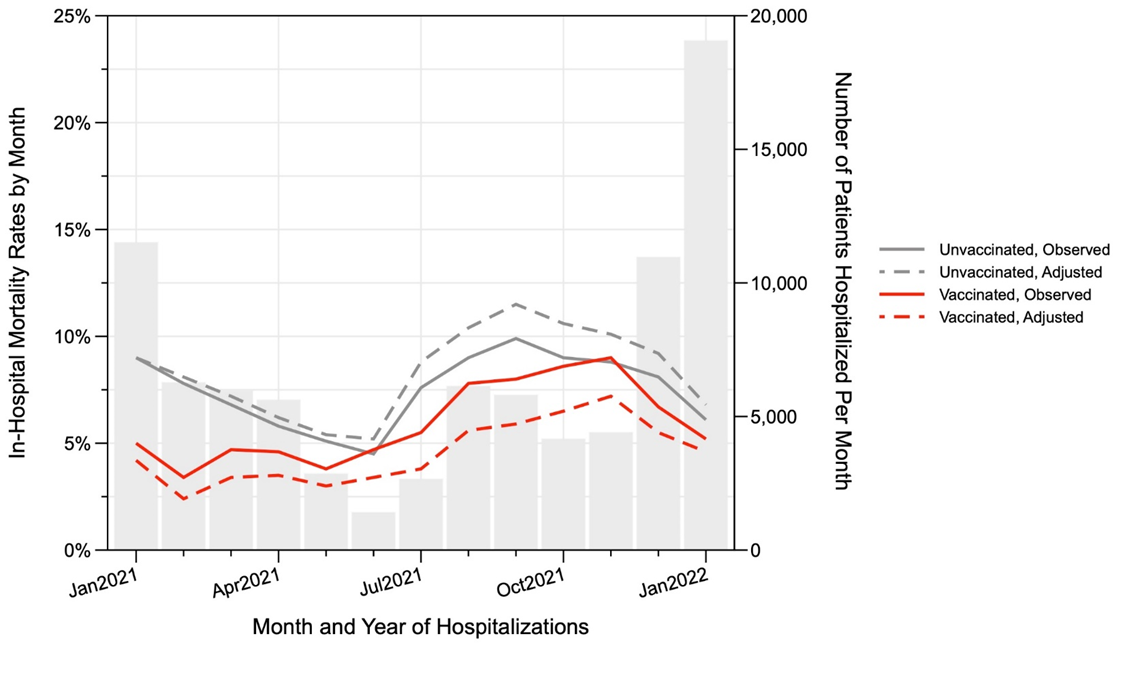


**S Figure10b** . Observed (unadjusted) mortality rates for patients who had received 0-3 COVID-19 vaccine doses (the continuous measure of vaccination status) over the study period along with rates of patients hospitalized per month.


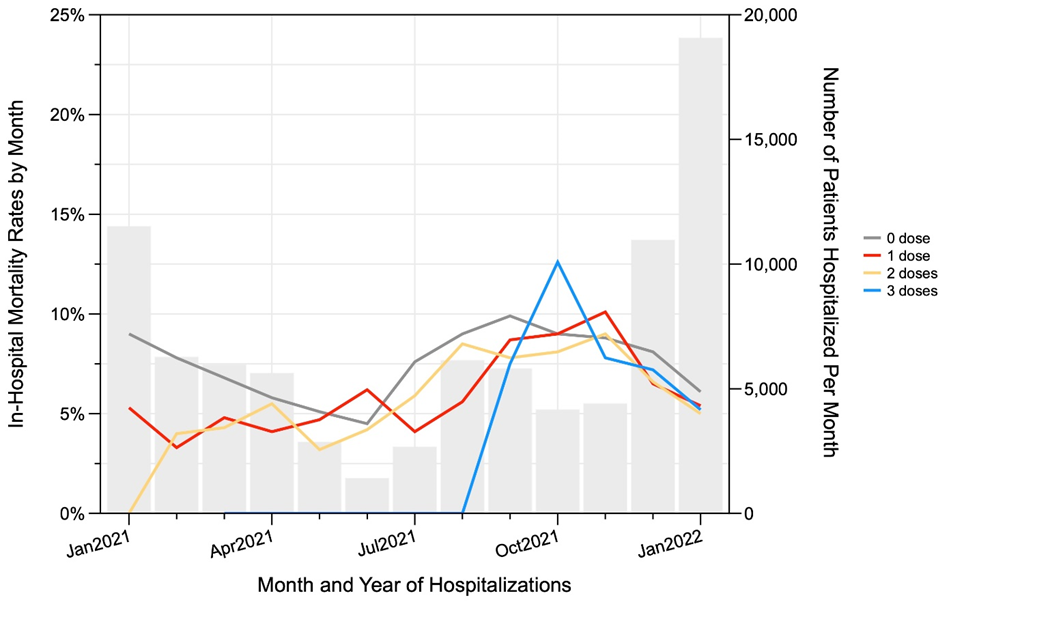


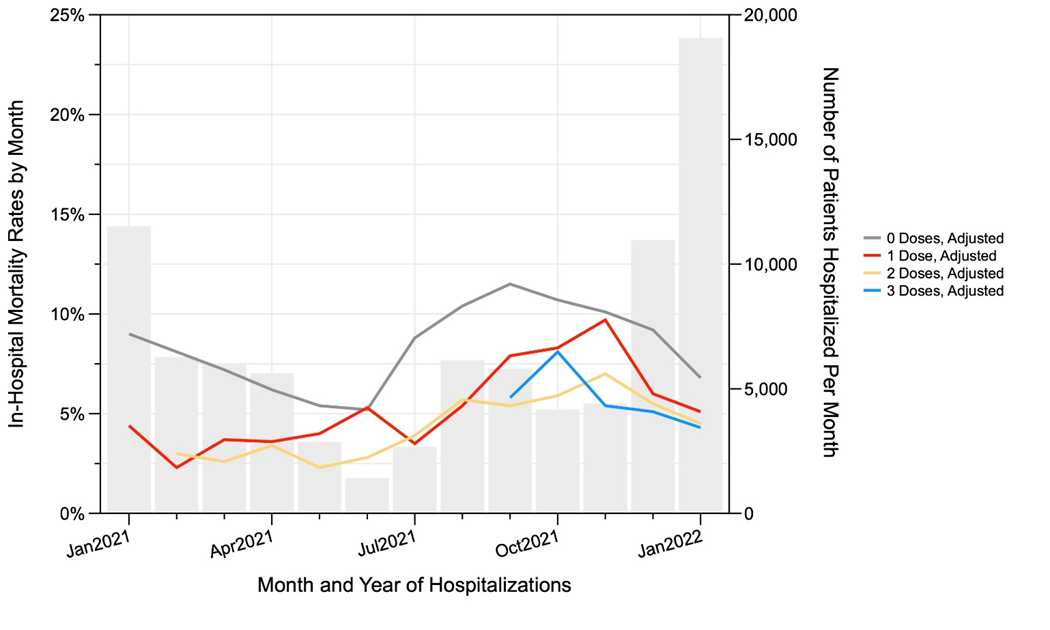
**S Figure 10c.** Adjusted mortality rates for patients who had received 0-3 COVID-19 vaccine doses (the continuous measure of vaccination status) over the study period along with rates of patients hospitalized per month.
